# Hard meets soft: tuning binary ferrofluids

**DOI:** 10.1039/d5nr05218a

**Published:** 2026-03-06

**Authors:** Malika Khelfallah, Ekaterina V. Novak, Andrey A. Kuznetsov, Deniz Mostarac, Niéli Daffé, Marcin Sikora, Sophie Neveu, Jovana Zečević, Johannes D. Meeldijk, Dario Taverna, Philippe Sainctavit, Mauro Rovezzi, Hebatalla Elnaggar, Enzo Bertuit, Nicolas Mille, Rachid Belkhou, Vincent Dupuis, Claire Carvallo, Amélie Juhin, Sofia S. Kantorovich

**Affiliations:** a IMPMC, CNRS UMR7590, Sorbonne Université, MNHN 4 Place Jussieu Paris France malika.khelfallah@gmail.com amelie.juhin@sorbonne-universite.fr; b Ural Federal University Lenin Av. 51 Ekaterinburg 620000 Russian Federation; c Faculty of Physics, University of Vienna Boltzmanngasse 5 1090 Vienna Austria sofia.kantorovich@univie.ac.at; d Sorbonne Université, CNRS, PHysicochimie des Électrolytes et Nanosystèmes InterfaciauX (PHENIX) F-75005 Paris France; e Synchrotron SOLEIL, L'Orme des Merisiers Saint-Aubin – BP48 91192 Gif-sur-Yvette France; f AGH University of Science and Technology, Academic Centre for Materials and Nanotechnology Al. Mickiewicza 30 30-059 Krakow Poland; g Inorganic Chemistry and Catalysis, Debye Institute for Nanomaterials Science, Utrecht University Universiteitsweg 99 3584 CG Utrecht The Netherlands; h European Synchrotron Radiation Facility (ESRF) 6 Rue Jules Horowitz BP220 38043 Grenoble Cedex 9 France; i Univ. Grenoble Alpes, CNRS, IRD, Irstea, Météo France, OSUG, FAME 38000 Grenoble France

## Abstract

We study binary ferrofluids composed of multicore “nanoflowers” of magnetically hard CoFe_2_O_4_ and magnetically soft MnFe_2_O_4_, as a way to optimise heat dissipation while suppressing aggregation–properties essential for biomedical applications. Bulk magnetometry and molecular dynamics simulations were combined to elucidate their behaviour. Experiments show wasp-waisted hysteresis, composition-dependent coercivity, and strong protocol dependence (field cooling). Simulations reproduce these trends and reveal the underlying structure–property coupling: (i) in an applied magnetic field, CoFe_2_O_4_ forms chains that dominate collective switching; (ii) adding MnFe_2_O_4_ “poisons” these chains—shortening and de-branching clusters—thereby lowering coercivity and loop area relative to a weighted superposition of the individual component responses without interactions; (iii) dipolar coupling reciprocally hardens the magnetically soft phase and softens the magnetically hard phase even without large-scale aggregation; and (iv) at higher total volume fraction (*ϕ* = 0.1) magnetically soft particles still suppress chain growth, reducing mean cluster size by up to an order of magnitude while keeping heating-relevant hysteresis close to Stoner–Wohlfarth expectations. These results establish composition-controlled microstructure as a means to decouple thermal output from aggregation: CoFe_2_O_4_ : MnFe_2_O_4_ mixtures can be tuned to enhance loss mechanisms while mitigating aggregation, offering a route to binary ferrofluids optimized for magnetic hyperthermia and drug delivery.

## Abstract 2

Binary ferrofluids of hard–soft magnetic nanoflowers show composition-controlled hysteresis and aggregation. Experiments and simulations reveal how soft particles suppress chain growth while preserving heating-relevant magnetic losses.

## Graphical abstract

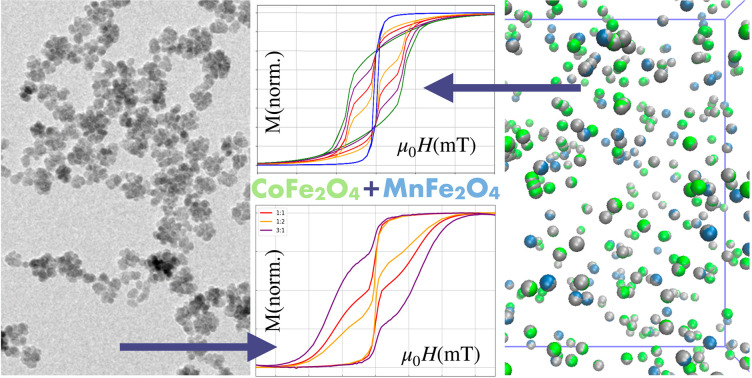

## 1. Introduction

Magnetic soft matter traces its origins to the early theoretical framework developed by Langevin, who first described the behaviour of non-interacting magnetic spins under an applied magnetic field.^1^ Building on these foundations, Elmore contributed crucial insights by showing that magnetic filings suspended in a liquid exhibit a Langevin-like magnetisation behaviour.^2^ It was the landmark work by Resler and Rosensweig in 1964 – reporting stable colloidal suspensions of magnetic nanoparticles, now commonly known as ferrofluids^3^ – that marked the emergence of magnetic soft matter as a distinct field of study. Since then, the field has expanded well beyond traditional ferrofluids^4–9^ to encompass a diverse range of magnetic soft materials such as magnetic gels and elastomers (polymer networks embedded with magnetic particles),^10–15^ filaments (polymer-like structures containing magnetic particles),^16–21^ and multicore particles (clusters of many magnetic cores within a single particle).^22–27^ Alongside this diversification, advances in chemical synthesis and particle engineering^28–30^ have enabled researchers to fine-tune the properties of magnetic soft materials and appropriate them for applications in technology,^31–33^ arts^34^ and biomedicine.^35–38^

However, polydispersity – variations in particle size, shape, or composition within a sample – poses a significant challenge. It alters the magnetic behaviour because particles of different sizes respond differently to magnetic fields. For example, studies on conventional ferrofluids have shown that smaller particles can interfere with the formation of long chains of larger particles.^39,40^ These chains^41–45^ form through magnetic dipole–dipole interactions and are critical to achieve a strong magnetic response. When smaller particles disrupt chain formation, the overall magnetic responsiveness of the fluid decreases.^46,47^ Another important factor influencing magnetic responsiveness is the size and structure of the particles themselves. Single-core magnetic nanoparticles consist of one magnetic domain.^48^ Multicore particles consist of several magnetic cores embedded within a larger matrix.^49^ Recent research has demonstrated that these multicore particles often show superior heating efficiency compared to their single-core counterparts.^50^ This is attributed to collective magnetic interactions within the multicore structure that enhance energy dissipation during magnetic excitation.

Beyond size and core number, magnetic anisotropy – the directional dependence of a particle's magnetic properties – also plays a crucial role in determining heat dissipation mechanisms.^51^ Magnetic anisotropy arises from factors such as particle shape, crystal structure, and surface effects, and dictates how easily the magnetic moment can reorient within the magnetic particle. Particles with high magnetic anisotropy (often referred to as “magnetically hard”) dissipate energy primarily through Brownian relaxation.^52^ This mechanism involves the mechanical rotation of the entire particle in response to the alternating magnetic field, generating heat through friction with the surrounding medium. Brownian relaxation tends to be most efficient at lower frequencies of the applied field. In contrast, “magnetically soft” particles with low anisotropy relax predominantly *via* the Néel mechanism,^53^ where the magnetic moment flips direction internally without the particle rotating. Néel relaxation is generally activated more effectively at higher frequencies, and its heating capacity is associated with internal magnetic losses rather than mechanical movement.

Each relaxation mechanism has distinct implications for biomedical use. Magnetically hard particles can generate significant heating but tend to aggregate because of strong magnetic interactions, limiting their suitability for such applications. Magnetically soft particles are less prone to aggregation, but typically generate less heat. Furthermore, safety limits, typically determined based on the Brezovich criterion,^54,55^ restrict the maximum product of the magnetic field amplitude and frequency used in treatments, placing practical constraints on heating efficiency. Given these considerations, mixing magnetically hard and soft particles presents a promising strategy to balance heating performance with safe and stable behaviour within the body. By combining particles that dissipate heat through complementary mechanisms, it may be possible to optimise both thermal output and transport properties, enhancing therapeutic outcomes.

Despite the potential of this approach, only very few binary ferrofluid systems have been experimentally studied so far. Most investigations have been limited to bulk magnetometry measurements, which only provide an average picture of the magnetic behaviour, and to transmission electron microscopy (TEM) images obtained from dried ferrofluids, whose particle organisation does not reflect that present in the liquid phase. Advanced methods based on electron/X-ray spectroscopy and microscopy allowing for chemical selectivity and/or preservation of the ferrofluid structure can be employed. However, such experiments are rare and challenging, largely due to the need for specific instruments or facilities (*e.g.* synchrotron facilities) and for dedicated sample environments and conditions (*e.g.*, liquid or cryogenic cells). So far, this has hindered the systematic experimental investigation of binary ferrofluids and a detailed understanding of their structural and magnetic properties – both of which are essential for identifying the key physical parameters needed for optimising binary ferrofluids for specific applications.

A number of experimental findings suggest that binary systems composed of CoFe_2_O_4_ (magnetically hard) and MnFe_2_O_4_ (magnetically soft) nanoparticles hold significant interest for advancing this question.^56^ In a zero-field-cooled 1 : 1 (volume ratio) binary ferrofluid composed of 6 nm spherical particles, element-selective magnetisation curves (*i.e.*, measured for each magnetic component) revealed the effects of magnetic dipolar interactions. Specifically, the magnetically hard phase exhibits a decrease in coercivity (*i.e.*, it becomes magnetically softer), whereas the soft phase shows an increase in coercivity (*i.e.*, it becomes magnetically harder^57^). This modification of magnetic properties takes place while particles remain isolated, without forming any structures like chains or clusters.

In this work, we systematically investigate how variations in particle magnetic anisotropy and fractional composition influence the magnetic response and heating efficiency of CoFe_2_O_4_–MnFe_2_O_4_ multicore (“nanoflower”) binary systems. We examine both frozen (solidified) and liquid samples to distinguish the roles of particle mobility and aggregation. The study is motivated by our experimental observations indicating a rich interplay between structural and magnetic properties in multicore CoFe_2_O_4_–MnFe_2_O_4_ mixtures, leading to intriguing physical behaviour possibly connected to the ability of CoFe_2_O_4_ nanoparticles to self-assemble into chains. As these results primarily serve as motivation, they are provided in the SI, with a short accompanying discussion presented below. Element-selective X-ray microscopy performed on slowly dried samples of binary mixtures shows that both particle types are embedded in large clusters and chains, suggesting strong magnetic interactions between them (Fig. S4). This structure differs markedly from that of a vitrified 1 : 1 mixture of CoFe_2_O_4_ (25 nm) and MnFe_2_O_4_ (15 nm), where the MnFe_2_O_4_ nanoparticles localize near chains formed exclusively by CoFe_2_O_4_ particles (Fig. S1). Complementary element-selective magnetometry measurements on a 1 : 1 mixture of CoFe_2_O_4_ (25 nm) and MnFe_2_O_4_ (20 nm) show that (i) under zero-field-cooling conditions, the coercivity of each component matches that of the corresponding monocomponent ferrofluid, although the magnetisation-curve shape is modified; and (ii) field cooling significantly increases the coercivity and remanence of the CoFe_2_O_4_ component within the binary mixture (Fig. S2). Identifying the mechanisms behind this process is experimentally challenging. Therefore, alongside experimental techniques such as magnetometry, we have used molecular dynamics (MD) simulations to investigate how nanoparticle assembly interacts with magnetic behaviour in binary mixtures.

## 2. Results and discussion

### 2.1. Experimental

**Fig. 1:**
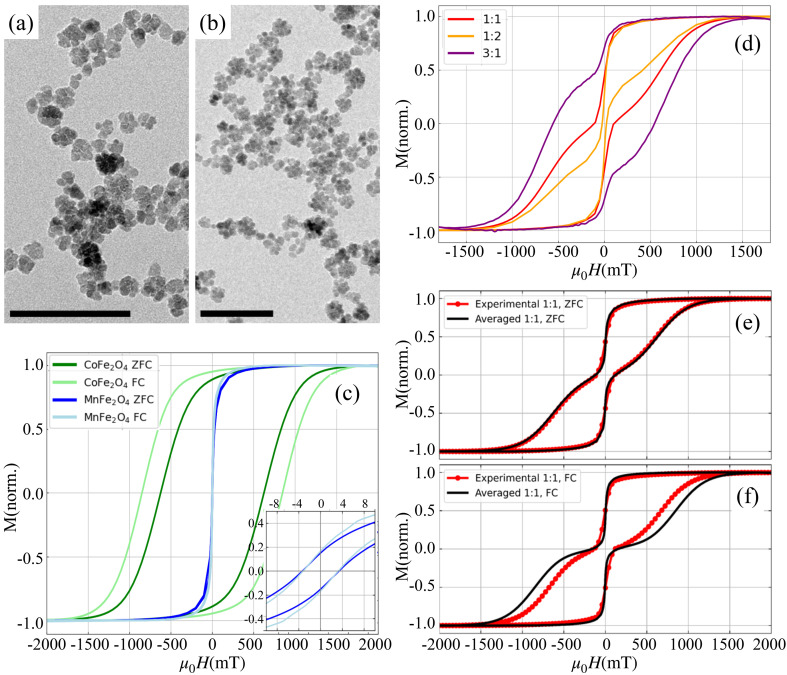
TEM images of samples (a) FF_Co_ and (b) FF_Mn_ (scale bar = 100 nm). (c) Magnetisation curves of FF_Co_ and FF_Mn_ samples measured at 100 K. Inset figure shows the zoomed in magnetisation in the vicinity of *H* = 0. (d) Magnetisation curves of the ZFC binary ferrofluids at different FF_Co_ : FF_Mn_ ratios measured at 100 K. (e) ZFC and (f) FC experimental magnetisation curves (red) and averaged magnetisation curves of 1 : 0 and 0 : 1 systems (black) measured at 100 K.

The binary mixtures studied in this work are composed of nanoflowers – permanent clusters of single domain magnetic cores, either CoFe_2_O_4_ (magnetically hard) or MnFe_2_O_4_ (magnetically soft). The nanoflowers are polydisperse with normal size distributions: CoFe_2_O_4_ have average diameter *d* = 15.8 ± 3.8 nm (Fig. S3(a)), and MnFe_2_O_4_ have average diameter *d* = 21.6 ± 7.4 nm (Fig. S3(b)). The nanoflowers are citrate-stabilised, dispersed in deionised water at a fixed volume fraction (*ϕ* = *V*_nanoflower_/*V*_total_ = 0.001). To assess the influence of mixing magnetically hard and soft phases, we analysed five systems: two monocomponent ferrofluids (FF_Co_ and FF_Mn_; see Fig. 1(a) and (b), respectively), a 1 : 1 volume ratio binary mixture, and two asymmetric mixtures with volume fraction ratios of CoFe_2_O_4_ to MnFe_2_O_4_ equal to 1 : 2 and 3 : 1. Binary ferrofluids were prepared by thoroughly mixing individual samples under controlled conditions to ensure homogeneity and prevent sedimentation or agglomeration.

Fig. 1(c) shows magnetisation curves *M*(*H*) of vitrified monocomponent ferrofluids: FF_Co_ exhibits high coercivity and remanence ratio *M*(*H* = 0)/*M*_s_, which is the ratio between the remnant magnetisation (*i.e.* the magnetisation in zero field after saturation) and the magnetisation at saturation (624 mT, 89%). In other words, FF_Co_ particles are magnetically hard. On the other hand, FF_Mn_ is magnetically soft, with a low coercivity and remanence ratio (3.4 mT, 15%). The FF_Mn_ sample remanence ratio of 15% is far below the theoretical values of 50% (for uniaxial anisotropy) or 83–86% (for cubic magnetocrystalline anisotropy) as reported by Usov *et al.*^58^ This discrepancy can be attributed to the high packing density within the clusters, which prevents coherent magnetic alignment. Similar behaviour has been observed in iron oxide nanoparticle powders, where increasing packing density reduces *M*(*H* = 0)/*M*_s_ to values as low as 18%.^59^ The remanence of FF_Co_, on the other hand, is consistent with cubic magnetocrystalline anisotropy. We can see that the cooling history affects the hysteresis loops. Namely, the hysteresis loop area is higher when the sample is cooled with an applied magnetic field (*i.e.* field cooled, FC, under 2T), as compared to the case where the sample is cooled without an applied magnetic field (*i.e.* zero field cooled, ZFC). This effect is attributable to an increased orientational ordering of dipoles within the nanoflower clusters.

Hysteresis curves of vitrified binary mixtures (Fig. 1(d)) show a wasp-waisted shape, indicating distinct switching fields for MnFe_2_O_4_ (low field) and CoFe_2_O_4_ (high field). Increasing the CoFe_2_O_4_ content raises both coercivity and loop area. Here, the hysteresis depends not only on the cooling history, but also on the ratio between the volume fractions of MnFe_2_O_4_ and CoFe_2_O_4_. The differences in the hysteresis loops are attributable to the changes in the microstructure and the inter-particle correlations in the system with the variation of the granulometric composition in the system. In 1 : 1 binary mixtures (Fig. 1(e) and (f), red curves), the FC sample requires a stronger field to remagnetise. In order to check the impact of interactions, experimental magnetisation curves can be compared to theoretical superpositions of individual FF_Co_ and FF_Mn_ responses:1
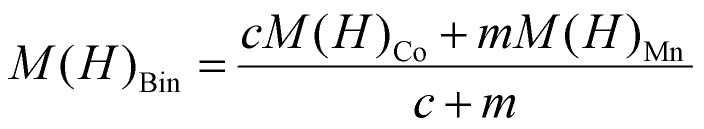
where *c* and *m* correspond to the proportions of FF_Co_ and FF_Mn_ in the binary mixture studied. These averaged theoretical curves are plotted as solid black lines in Fig. 1(e) and (f). In this formulation, the two components (FF_Co_ and FF_Mn_) do not interact with each other.

Magnetisation curves *M*(*H*) measured in binary ferrofluids in the FC configuration show an increase in coercivity and remanence compared to the ZFC configuration. However, due to the bulk measurement nature of SQUID magnetometry, it remains challenging to attribute these changes in coercivity or remanence specifically to either FF_Co_ or FF_Mn_ nanoflowers. To elucidate these modifications in the magnetic properties of binary ferrofluids, observing the assemblies formed by the nanoparticles in the ferrofluids is essential. In this context, coarse-grained MD simulations provide a cost-effective and scalable way to study binary mixtures. *In silico*, one can visualise and perform structural analysis of binary ferrofluids under bulk-like conditions, offering access to statistically robust data and parameter sweeps that are difficult or prohibitively expensive to achieve experimentally. Simulations also allow one to probe time-resolved dynamics and field-induced organisation with a level of control and reproducibility that complements and extends the insight gained from experimental observations.

### 2.2. Computational

Magnetic nanoparticles (MNPs) are often modelled as particles with point dipoles, fixed in the particle body-centred reference frame, undergoing only Brownian relaxation.^60–62^ Such a modelling approach is only valid for magnetically hard particles (magnetic anisotropy energy is at least an order of magnitude higher than thermal energy). Magnetically soft (magnetic anisotropy energy is similar to thermal energy) colloids exhibit several additional internal relaxation mechanisms, the most prominent of which is the Néel relaxation,^53^ as noted previously. To be able to consider the phenomenology associated with internal relaxation mechanisms of magnetically soft colloids *in silico*, we use two advanced approaches,^21,63^ which we refer to as the ideally magnetisable superparamagnet model and the egg model. In both cases, we exclusively model a uniaxial magnetic anisotropy that can be characterised by a single effective anisotropy constant *κ* = *KV*/*k*_B_*T*, with *K* being the material anisotropy constant, *V* – particle volume and *k*_B_*T* denoting thermal energy. In the first approach, the magnetic anisotropy energy of magnetic particles is considered negligible, *κ* ∼ 0, and the instantaneous magnetic response to a magnetic field is determined based on the Langevin function^1^ of the total magnetic field (vectorial sum of the applied and dipole fields) acting on the particle. The second approach combines translation and rotational Brownian dynamics of particles with magnetisation dynamics of their dipole moments described *via* the overdamped Landau–Lifshitz–Gilbert (LLG) equation.^64^ More details on both models are provided in the Methods section.

Binary mixtures were simulated at a fixed total volume fraction of *ϕ* = 0.001, while the volume fraction of MnFe_2_O_4_, denoted *ϕ*_s_, was varied as *ϕ*_s_ = 0, 0.00025, 0.00033, 0.0005, 0.00067, 0.00075, 0.001 (corresponding to CoFe_2_O_4_ : MnFe_2_O_4_ ratios from 1 : 0 to 0 : 1). We use *ϕ*_s_ to label the MnFe_2_O_4_ fraction, facilitating comparison between experimental and simulated systems. The simulated systems are referred to as point-dipole-egg (PDE) systems. Particle sizes and interactions were taken from experimental averages.

**Fig. 2:**
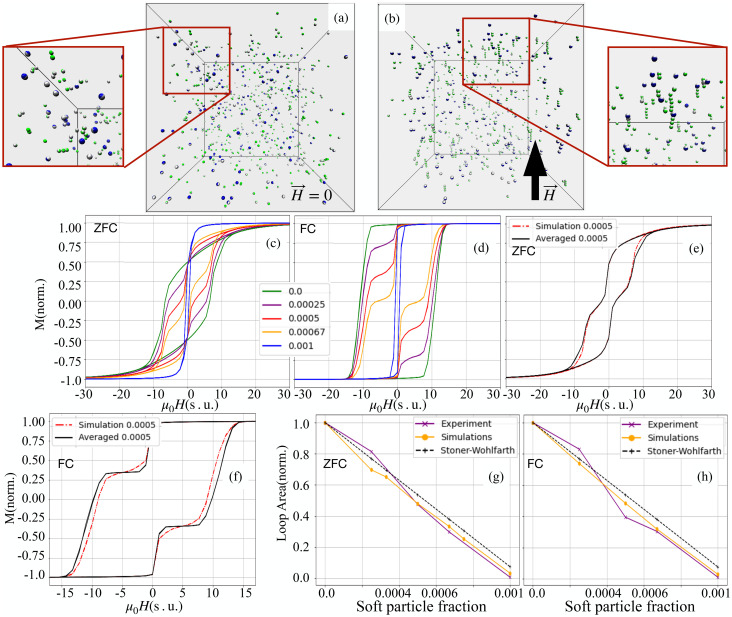
(a and b) Typical room-temperature snapshots of the PDE 1 : 1 mixture at *ϕ* = 0.001: (a) without field and (b) under a saturating field. Green and blue spheres represent CoFe_2_O_4_ and MnFe_2_O_4_ nanoparticles, respectively; grey hemispheres indicate the internal magnetisation vector, orthogonal to the plane dividing the coloured and grey halves. Insets show zoomed-in clusters. (c–f) Magnetisation curves of frozen binary ferrofluids: (c) ZFC case, for comparison with Fig. 1(d); (d) FC case. Legends indicate *ϕ*_s_ values. (e) ZFC magnetisation for the 1 : 1 sample (red dash-dot) with the superposition from eqn (1) (black); compare with Fig. 1(f). (f) Same as (e) for the FC case. (g and h) Areas of the hysteresis loops normalised by the largest area for a monodisperse CoFe_2_O_4_ case: experiment (purple), simulations (orange), and the ideal-mixture Stoner–Wohlfarth model (eqn (3), black dashed line). (g) ZFC and (h) FC cases.

Our analysis of the binary systems begins with modelling thermodynamic equilibrium at room temperature. Under these conditions, both CoFe_2_O_4_ and MnFe_2_O_4_ particles are represented as single point dipoles. For CoFe_2_O_4_, the dipole moment is fixed within the particle body, allowing only Brownian relaxation, whereas for MnFe_2_O_4_, the dynamics of the dipole moment evolve according to the egg model (described in the Methods section). Fig. 2(a) and (b) show representative equilibrium (room temperature) simulation snapshots of the PDE 1 : 1 mixture of CoFe_2_O_4_ (green) and MnFe_2_O_4_ (blue) nanoparticles. In panel (a), without an external field, the nanoparticles are isotropically distributed. Panel (b), under a saturating magnetic field, shows alignment and chaining of anisotropic CoFe_2_O_4_ particles, while MnFe_2_O_4_ ones remain mainly nonaggregated with partial moment alignment. This structural reorganisation under an applied field highlights the distinct magneto-responsive behaviour of the two nanoparticle species within the binary colloidal suspension.

From the magnified views in Fig. 2(a) and (b), one can deduce a typical cluster topology and the particle positioning within them. In the absence of an external magnetic field (Fig. 2(a)), the magnified view reveals that MnFe_2_O_4_ either remains single or forms compact clusters (see the bottom-right corner of the magnified view in Fig. 2(a)). CoFe_2_O_4_ nanoparticles, instead, are arranged in short, chain-like structures, often extending across several particle diameters. In some cases, a MnFe_2_O_4_ attaches to the end of such a chain (see the left side of the magnified view in Fig. 2(a)). Comparing across the magnified views of Fig. 2(a) and (b) (no field and saturating field conditions, respectively), one can see a change in particle arrangements. CoFe_2_O_4_ chains become longer and align along the field direction, with individual particle moments closely following the field vector. This reflects the dominance of Zeeman energy over thermal fluctuations for high-anisotropy (*κ* ≫ 1) particles. Meanwhile, MnFe_2_O_4_ moments also align with the field, but they remain nonaggregated to a large extent. Notably, in the chain structures, MnFe_2_O_4_ particles are found mainly at the edges, rather than in-between CoFe_2_O_4_ particles. This indicates that the combined effect of the local dipolar and external fields enhances their effective anisotropy, reducing magnetisation flips that would otherwise break the chains. These simulation results qualitatively agree with cryo-TEM observations of vitrified samples (Fig. S4). However, the simulated clusters are smaller and less developed. This discrepancy likely stems from (i) the fact that simulated colloids are perfectly stable (no effective central attraction, typically present in experimental systems as a manifestation of hydrophobicity/van der Waals interactions, particularly during vitrification) and (ii) due to the non-instant cooling in the experiment.

To justify the model and confirm that our model captures magnetic interactions correctly, we froze 50 independent simulation configurations instantly for each system and performed multiple slow magnetisation cycles to obtain hysteresis loops. Importantly, this *in silico* experiment accounts for the finite magnetic anisotropy of CoFe_2_O_4_ – although over six times magnetically harder than MnFe_2_O_4_, a saturating slow field in a frozen sample can flip its magnetisation once the coercivity threshold is exceeded. In this part of the analysis, the hysteresis loops were simulated at *T* equivalent to 100 K, used in the experiment. Slower cooling in simulations would suppress entropic contributions to the free energy, thereby promoting stronger aggregation, which is expected to enhance chain growth, favour the formation of larger and more branched structures, as observed previously in conventional ferrofluids.^65^ The poisoning by soft particles will still be present in the system.

Fig. 2(c) and (d) display normalised magnetisation as a function of the applied field (in simulation units, see Methods for details) under zero field-cooled (ZFC) and field-cooled (FC) protocols, respectively. As the volume fraction *ϕ*_s_ increases, the magnetisation curves of ZFC samples become progressively narrower, and the hysteresis diminishes. In the case of FC cooled samples, the loops are much broader. Interparticle interactions, enhanced by prealignment, strongly affect coercivity, locally increasing the anisotropy of MnFe_2_O_4_. This observation is supported by the curves in Fig. 2(e) and (f), that offer a direct comparison between simulated data (red) for the 1 : 1 mixture and the superposition of magnetisation loops for pure CoFe_2_O_4_ and MnFe_2_O_4_ components, calculated from eqn (1). The difference between these two sets of curves is strongly pronounced under FC conditions. Fig. 2(c) should be compared with Fig. 2(d), where the colour coding for different mixing ratios of CoFe_2_O_4_ and MnFe_2_O_4_ particles is consistent across both plots. The overall agreement between simulation and experiment is encouraging, both qualitatively and quantitatively. The key distinction lies in the shape of the hysteresis loops: in the experimental data, a more pronounced saturation plateau is observed, whereas in the simulations, the magnetisation increases and decreases more gradually with the applied field.

This discrepancy can be attributed to several factors. Firstly, the experimental system exhibits polydispersity in particle size and magnetic properties, which is not captured in the simulation model. In particular, size polydispersity inevitably leads to a distribution of effective magnetic anisotropies, due to the combined influence of volume, surface, and shape anisotropy contributions, thereby modifying the energy barrier landscape for magnetisation reversal. Secondly, the real CoFe_2_O_4_ and MnFe_2_O_4_ particles are multicore structures, which can exhibit collective magnetic behaviour not present in the simplified simulation particles. Even a small fraction of large, highly magnetic CoFe_2_O_4_ particles can dominate the magnetic response, contributing to the more square-like shape and sharper switching observed in the experimental hysteresis loops. A similar type of deviation is observed when comparing Fig. 2(e) and (f) with their experimental counterparts in Fig. 2(e) and (f). In all four plots, the magnetisation for the ZFC (panels (e)) and FC (panels (f)) cases is shown in red, while the black curves are obtained in the no-interaction assumption from eqn (1). While the simulations tend to show a smoother magnetisation response compared to the experimental data – consistent with the previously discussed effects of particle polydispersity and multicore structure – the key trends are closely reproduced.

In order to strengthen the statement above, in Fig. 2(g) and (h), we show the normalised magnetic hysteresis loop area as a function of magnetically soft particle volume fraction, *ϕ*_s_, in binary mixtures of magnetic particles. The hysteresis loop area *A* in general can be calculated as:2
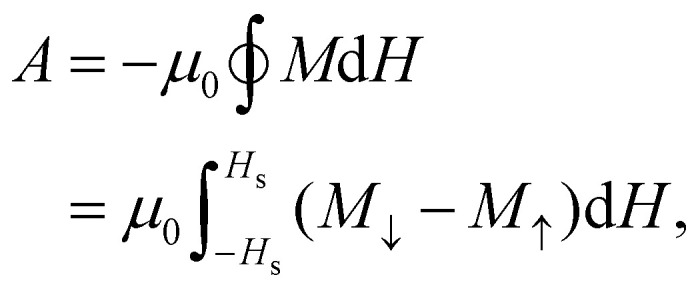
where 
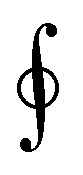
 indicates the integration over the full cycle of the field alteration, *H*_s_ is the saturation field value, *M*_↓_ = *M*_↓_(*H*) is the descending upper branch of the loop (*i.e.*, the magnetisation curve measured from +*H*_s_ to −*H*_s_), *M*_↑_ = *M*_↑_(*H*) is the ascending lower branch. The binary Stoner–Wohlfarth model (see Methods for details) assumes a linear superposition of anisotropy contributions from the two components and gives for the normalized loop area the expression:3
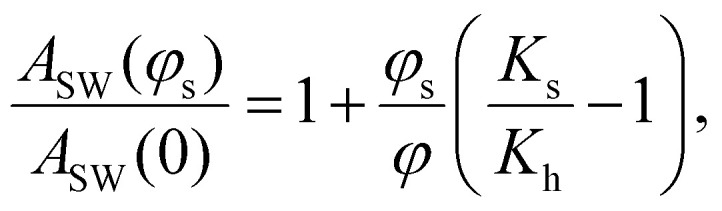
where *K*_h_ and *K*_s_ are the material anisotropy constants of magnetically hard and soft particles respectively. This model (3) is consistently above both simulations and experiments, likely due to interparticle interactions and structural effects not included in the analytical approximation.

**Fig. 3:**
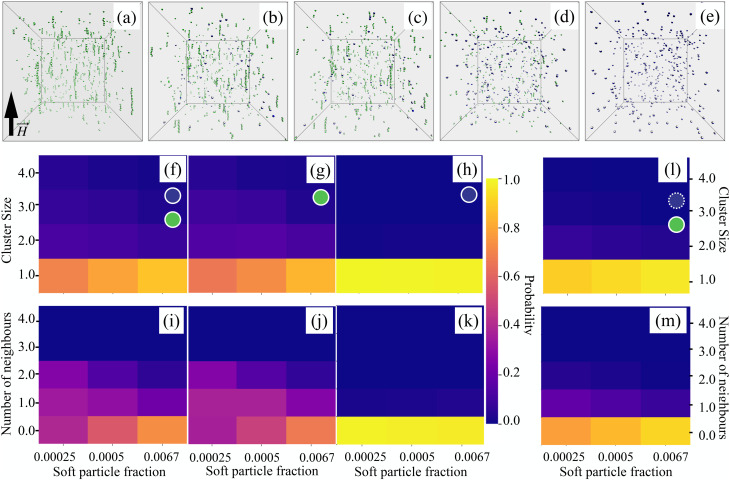
(a–e) Typical room-temperature snapshots under a saturating magnetic field (field direction indicated on the left). The total volume fraction is 0.001. Configurations correspond to CoFe_2_O_4_ : MnFe_2_O_4_ ratios of (a) 1 : 0, (b) 3 : 1, (c) 1 : 1, (d) 1 : 2, and (e) 0 : 1. Green nanoparticles represent CoFe_2_O_4_, and blue nanoparticles represent MnFe_2_O_4_. (f–h) Heat maps showing the probability of finding clusters of specific sizes at different values of the magnetically soft particle volume fraction, under a saturating magnetic field: (f) both particle types in the mixtures are considered (PDE); (g) only magnetically hard particles are considered; (h) only magnetically soft particles are considered. (i–k) Heat maps of the probability for a particle to have a given number of nearest neighbours at different volume fractions, under a saturating magnetic field: (i) both particle types in the mixtures are considered (PDE); (j) only magnetically hard particles are considered; (k) only magnetically soft particles are considered. Panels (f) and (i) display results for all particles (both types), while panels (g) and (j) show only magnetically hard particles, and panels (h) and (k) only magnetically soft particles, as indicated by the green and blue circles, respectively. (l and m) (l) Probability of finding clusters of specific sizes, and (m) probability of a particle having a given number of nearest neighbours, at different volume fractions under a saturating magnetic field for mixtures of ideally superparamagnetic (blue, dashed outline) and magnetically hard (green) particles. Each plot is based on 50 statistically independent simulation snapshots. Clusters were identified using an energy-distance criterion: two particles belong to the same cluster if their separation is no more than 30% greater than the close-contact distance and if their dipolar interaction is attractive (*i.e.*, negative).^61^

Fig. 3(a)–(e) show representative snapshots of the equilibrium state of binary PDE mixtures, illustrating the gradual transition from a pure CoFe_2_O_4_ system (green) to a pure MnFe_2_O_4_ system (blue), passing through intermediate 3 : 1, 1 : 1, and 1 : 2 compositions. The orientation of each particle's magnetic dipole is indicated by the grey hemispheres. As the composition shifts from left to right across the panels, the extent of particle aggregation steadily diminishes. Notably, in Fig. 3(c) and (d), where the system is more evenly mixed, the blue, magnetically soft particles, tend to associate with the green, magnetically hard ones. Clusters are primarily composed of magnetically hard particles and most frequently appear as linear chains.

To better quantify these observations, we examined the probability of finding clusters of a particular size (Fig. 3(f)–(h)) and the average number of nearest neighbours per particle (Fig. 3(i)–(k)). To highlight the specific behaviour of each particle type, we also performed analyses in which one type was excluded. Overall, all of the systems studied here aggregate weakly. The average cluster size decreases with increasing *ϕ*_s_, and the aggregation is driven by the CoFe_2_O_4_ particles. In a linear chain, internal particles have two neighbours, while end particles have one. If a particle has more than two neighbours, it represents a branching point; if it has none, it is isolated. It is that the probability of both branching and the formation of long chains is low, and declines on the increase of magnetically soft particle fraction. The overall aggregation becomes negligible without an applied magnetic field, as it is shown in a similar set of heat-maps in SI Fig. S5 and S6. Interestingly, if we artificially decrease the anisotropy energy of magnetically soft particles, *KV*, to zero (using the aforementioned ideally magnetisable superparamagnet model; see Methods section for details), even a stronger decrease in the clustering of magnetically hard particles clusters is observed (Fig. 3(l)) and the branching is fully suppressed (Fig. 3(m)).

The preferential attachment of MnFe_2_O_4_ particles to the ends of CoFe_2_O_4_ chains can be understood as a result of the competition between energetic and entropic contributions. Chain formation is generally entropically unfavourable but energetically stabilised by dipolar interactions. In this context, hard–hard particle bonds are energetically stronger (*i.e.* have a larger absolute interaction energy) than hard–soft or soft–soft bonds. Considering a minimal three-particle chain composed of two hard and one soft particle, placing the soft particle between two hard ones leads to two hard–soft bonds, whereas positioning the soft particle at the end of a hard–hard pair results in one hard–hard and one hard–soft bond, which is energetically more favourable. In addition, end attachment of the soft particle carries an entropic advantage, as it allows for a larger number of accessible configurations compared to incorporation within the chain. Once attached to the end of a hard-particle chain, the soft particle effectively poisons further chain growth, since the attachment of an additional hard particle to a soft one is energetically less favourable than hard–hard bonding. As the fraction of soft particles increases, the probability that both ends of a hard-particle chain become terminated by soft particles correspondingly increases, thereby suppressing further chain elongation.

We observed that even a small degree of aggregation already has a marked influence on the magnetic response, suggesting that it would be highly interesting to explore systems with higher particle concentrations. Experimentally, however, such studies are both costly and time-consuming, whereas in simulations they are readily achievable. Having now validated our model, we can confidently employ it to investigate these denser systems. To this end, we performed a series of *in silico* experiments in which the particle volume fraction *ϕ* of the PDE mixtures was increased by a factor of 100.

**Fig. 4:**
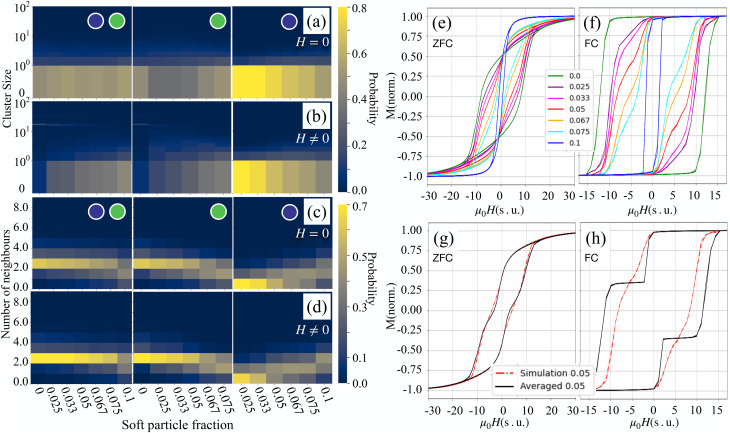
Probability heat maps showing cluster statistics in binary mixtures of magnetic particles at varying volume fractions of magnetically soft particles. The total particle volume fraction is fixed at 0.1 in all cases. Panels (a) and (b) display the probability of observing clusters of a given size in zero external field and under a saturating magnetic field, respectively. The *y*-axis is logarithmic. Panels (c) and (d) show the probability of finding particles with a specific number of neighbours, again in zero field and in a saturating field. In each of these panels, the left subpanel corresponds to the calculations in which both types of particles in the mixtures are considered (PDE); middle – only magnetically hard particles in mixtures are considered; and the right subpanel shows the results when only magnetically soft particles in mixtures are considered. Panels (e) and (f) present normalised magnetisation curves of the binary ferrofluids under zero field-cooled (ZFC) and field-cooled (FC) conditions, respectively, for various magnetically soft particle fractions. Panels (g) and (h) show comparisons of the simulated ZFC and FC magnetisation curves (red dashed-dotted line) for a 1 : 1 mixture with predictions from the linear superposition model described in eqn (1) (black line).

Fig. 4(a) and (b) illustrate how cluster size distributions vary with increasing magnetically soft particle content, both in the absence of an external magnetic field and under the influence of a saturating field. In zero field (Fig. 4(a)), for a total volume fraction *ϕ* = 0.1, the probability of finding an isolated (non-clustered) particle does not exceed 60%. In contrast, at a much lower concentration (*ϕ* = 0.001), at least 80% of particles remain non-aggregated. Interestingly, even in magnetically soft-particle-dominated systems, for *ϕ*_s_ > 0.033, nearly 30% of particles are observed to form dimers, indicating the onset of aggregation at higher concentrations. In Fig. 4(b), where a saturating magnetic field is applied, large clusters–containing up to 20 particles – are seen in mixtures with a low fraction of magnetically soft particles, with a probability exceeding 30%. As the proportion of magnetically soft particles increases, the likelihood of forming such large aggregates sharply decreases. This suggests that magnetically soft particles suppress clustering. Notably, unlike what we have seen in Fig. 3, where MnFe_2_O_4_ particles do not form clusters even at high fractions, the increased overall particle concentration in Fig. 4 allows for some limited aggregation among the magnetically soft particles as well.

Panels (c) and (d) of Fig. 4 further characterise the internal structure of these aggregates by showing the number of nearest neighbours per particle under the same conditions. At low magnetically soft particle fractions, particles – particularly the magnetically hard ones – tend to have more neighbours, indicating not only the presence of linear chains but also branched structures. For example, at *ϕ*_s_ = 0.05, around 10% of CoFe_2_O_4_ particles have three or more neighbours. In contrast, the fraction of magnetically soft particles serving as branching points never exceeds 10%, even in systems composed entirely of MnFe_2_O_4_ (*ϕ*_s_ = 0.1). When a saturating field is applied (Fig. 4(d)), the nearest neighbour distributions become narrower, with the two-neighbour configuration becoming dominant across all magnetically soft particle fractions. This reflects the enhanced alignment and regularity of chains under the influence of the external field. At high magnetically soft particle fractions (above 0.067), at least 30% of magnetically soft particles have exactly one neighbour, evidencing their participation in clustering as chain ends, but not in branching.

The magnetisation curves in panels (e) and (f) of Fig. 4 depend on the mixture composition. In zero field-cooled (ZFC) measurements, Fig. 4(e), the slope of magnetisation curves at zero field increases when *ϕ*_s_ decreases. However, compared to lower overall concentrations (see, Fig. 2(c)), interparticle interactions at *ϕ* = 0.1 significantly influence coercivity – the loops are notably wider. In field-cooled (FC) curves, Fig. 4(f), mixtures rich in magnetically hard particles show more abrupt magnetisation switching, indicating the presence of strongly interacting, collectively responding clusters. As the magnetically soft particle fraction increases, these loops become smoother and less hysteretic, consistent with reduced clustering and weaker collective behaviour.

**Fig. 5:**
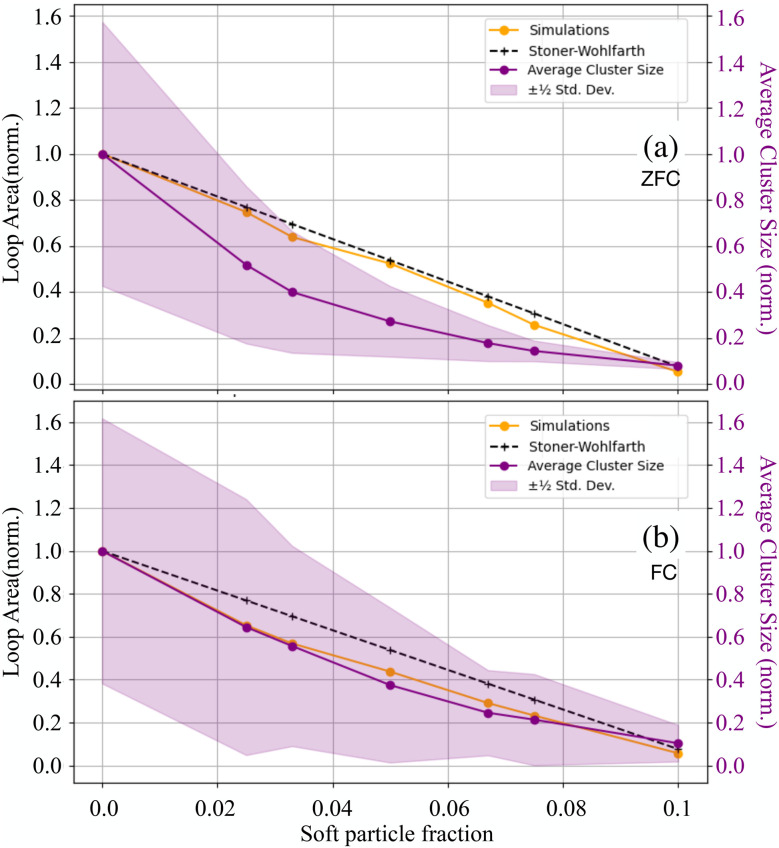
Normalised hysteresis loop area as a function of magnetically soft particle volume fraction for two preparation protocols: (a) ZFC and (b) FC. Simulation results (orange), Stoner–Wohlfarth model predictions (black dashed), and average cluster size (purple, right vertical axes) are shown. The shaded region around the cluster size indicates the standard deviation.

Panels (g) and (h) in Fig. 4 compare the simulated magnetisation curves of a 1 : 1 mixture to the predictions of a simple superposition model based on eqn (1). At this overall concentration, the interactions between magnetically soft and hard particles become significant – particularly in the FC case. The results indicate that magnetically soft particles are “hardened” through their interactions with the magnetically hard component. Additional evidence for the latter can be found in Fig. S7 in the SI. To further explore this effect, the simulations were performed in which interparticle interactions were disabled during the magnetisation loop calculations in the frozen state (see SI Fig. S8). These simulations confirm that interparticle interactions enhance the effective anisotropy of the magnetically soft component. At the same time, by suppressing clustering among magnetically hard particles, the magnetically soft component effectively reduces the overall coercivity of the mixture. This can be seen in Fig. 4(h), where the coercivity of the fully interacting system is notably lower than that predicted by the non-interacting model. Thus, magnetically soft particles simultaneously harden while softening the magnetically hard component – a dual role emerging from their interaction-driven influence on the microstructure. In Fig. 5, the normalised magnetic hysteresis loop area is contrasted with the normalised mean cluster size as a function of the magnetically soft particle volume fraction for (a) ZFC and (b) FC cases. Normalisation refers to dividing each curve by the respective value at *ϕ*_s_ = 0. The simulation-derived loop area (orange line) consistently decreases as the magnetically soft particle fraction rises, reflecting a diminished heat production. The theoretical prediction from the Stoner–Wohlfarth model for binary mixtures is represented by a dashed black curve, assuming non-interacting particles that are (a) randomly oriented or (b) completely aligned with the field. The normalized reduction in average cluster size, depicted as a purple line, includes fluctuations represented by shaded regions indicating standard deviations.

In a ZFC case, the mean cluster size, provided as the first line of Table 1 rapidly decreases, and so does the width of the cluster size distribution. Basically, while the loss in the magnetisation loop area is only on the order of 20% for *ϕ*_s_ = 0.02, the actual cluster size reduces by a factor of two. Therefore, the addition of a magnetically soft phase to a magnetically hard one is a viable and effective strategy to mitigate the risk of large aggregate formation when using magnetic fluids for *in vivo* hyperthermia. Even though the interactions are leading to a significant change in the structural properties, they are not reflected in the ZFC magnetisation loop area (heat production): the orange and the black curves are very close. This is due to the random orientation of the formed clusters in the bulk system.

**Table 1:** Average cluster size for zero field-cooled (ZFC) and field-cooled (FC) systems at various magnetically soft particle volume fractions *ϕ*_s_; *ϕ* = 0.1

*ϕ*_s_	0.0	0.025	0.033	0.05	0.067	0.075	0.1
ZFC	35.5	18.4	14.2	9.7	6.3	5.1	2.8
FC	38.2	24.6	21.3	14.3	9.4	8.2	3.9

In contrast, for the FC system, the orange curve is lower than a non-interacting Stoner–Wohlfarth approximation and follows the decay in the relative average cluster size. In the FC case, the clusters are predominantly aligned with the direction of the applied magnetic field, creating a strong anisotropic magnetic pattern. This pattern manifests itself in a strong deviation of the loop area from a non-interacting assumption. At the same time, the average cluster size in the FC case is 10% larger than in a field-free case (compare 35.5 and 38.2, Table 1, first column), but the poisoning, caused by MnFe_2_O_4_ particles, results in a 10-time drop of the cluster size (from 38 to 3.9 as seen in the lower row of Table 1), pushing the magnetisation closer to the Stoner–Wohlfarth result for the non-interacting particles. Interestingly, for *ϕ*_s_ = 0.033, the cluster size in a ZFC case reduces by 60%, but in the FC case – by 40% only. Regardless, this is still a major reduction in the maximal the cluster size and a narrowing of the distribution also in the FC case.

## 3. Conclusion

This study demonstrates the potential of engineering binary magnetic fluids through the combination of magnetically hard CoFe_2_O_4_ and soft MnFe_2_O_4_ nanoparticles to achieve tunable magnetic and structural responses, desirable for biomedical applications. By systematically investigating the influence of magnetic anisotropy, particle composition, and external field conditions, we show how binary systems exhibit emergent behaviour not observed in their monocomponent counterparts.

Magnetically hard CoFe_2_O_4_ nanoparticles tend to form chain-like structures, while MnFe_2_O_4_ ones barely participate in clustering. In binary mixtures, these contrasting behaviours give rise to partial structural ordering, particularly under field-cooled conditions. We have shown that binary mixtures exhibit wasp-waisted loops (Fig. 1d) and, critically, *are not* identical to a simple superposition of the single-component curves (eqn (1) and Fig. 1e, f). This difference is amplified under the field cooling conditions. Adding a magnetically soft phase lowers the effective coercivity of the magnetically hard phase, while the hard phase raises the effective anisotropy of the soft phase—exactly the “mutual hardening/softening” needed to reconcile heating with stability. Even though the magnitude of this effect is system specific, it will be present in any hard–soft particle mixture.

The integration of cryo-TEM imaging, MPMS magnetometry, and element-specific RIXS-MCD and STXM provides experimental evidence of these structural and magnetic phenomena, while MD simulations offer further insight into the role of intrinsic magnetic anisotropy and field strength. With the help of MD simulations, we were able to explain that with a magnetic field applied, CoFe_2_O_4_ drives chaining while MnFe_2_O_4_ inserts mainly at chain ends, suppressing branching points and shortening chains (“poisoning”). At fixed total volume fraction *ϕ* = 0.1, the mean cluster size drops from ∼38 (magnetically hard-only, FC) to ∼4 at *ϕ*_s_ = 0.1 (Table 1), and even a modest magnetically soft particle fraction yields large structural gains: at *ϕ*_s_ = 0.033 the average size decreases by ∼60% (ZFC) and ∼40% (FC), while the ZFC loop area remains close to the Stoner–Wohlfarth expectation for non-interacting mixtures (Fig. 5a), *i.e.* minimal drop in the hysteresis loop area (directly proportional to the magnetic heating power) despite major aggregation suppression.

The areas of FC loops fall *below* the non-interacting Stoner–Wohlfarth line and track the interaction-controlled reduction in cluster size (Fig. 5b), directly linking microstructure to heat dissipation. Our data show that composition-controlled poisoning in magnetically hard–soft nanoflower binary mixtures provides a practical route to “best-of-both-worlds” design: (i) minimal cluster formation for flow safety and low embolic risk, (ii) preserved (ZFC) or deliberately shaped (FC) hysteresis for efficient losses, and (iii) an interaction-enabled lever—mutual hardening/softening—available even *before* large particle clusters form.

Ultimately, this work highlights the versatility of composite ferrofluids comprising CoFe_2_O_4_ and MnFe_2_O_4_, and underscores the importance of coupling experimental techniques with advanced simulation approaches. The ability to tailor magnetic properties through particle selection and external field conditioning opens promising pathways for the design of responsive magnetic materials, particularly in biomedical applications such as hyperthermia, magnetic targeting, and sensing.

## 4. Methods

### 4.1. Experimental

#### 4.1.1. Synthesis and sample preparation

Cobalt ferrite nanoflowers, FF_Co_, were synthesized using the polyol process,^66^ which involves the forced hydrolysis of a mixture of Fe^3+^ and Co^2+^ ions in a polyol solution. The morphology of the resulting nanoparticles depends on the polyol used during synthesis. Specifically, when the reaction is conducted in a mixture of diethylene glycol (DEG) and *N*-methyl diethylamine (NMDEA) in a 50 : 50 *V* : *V* ratio, nanoflower-shaped particles are obtained. To ensure a stable colloidal suspension at pH = 7, the surface of the magnetic nanoparticles is functionalized with citrate molecules.^67^ The same process was used to synthesize the MnFe_2_O_4_ nanoflowers.

Binary ferrofluids were prepared by diluting the single-phase ferrofluids FF_Co_ and FF_Mn_ to a nanoparticle volume fraction of 0.1%. The binary samples were then created by mixing the diluted ferrofluids in specific volume ratios, ensuring that the total volume fraction of nanoparticles remained at 0.1%. This volume fraction, defined as
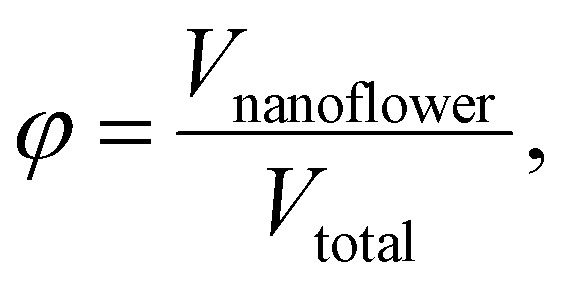
was chosen to promote self-assembly through magnetic dipolar interactions, as evidenced in prior studies, and to ensure sufficient signal strength for magnetometry measurements. The prepared binary ferrofluid ratios included 1 : 1 (equal volumes of FF_Co_ and FF_Mn_), 1 : 2 (one part FF_Co_ to two parts FF_Mn_), and 3 : 1 (three parts FF_Co_ to one part FF_Mn_).

#### 4.1.2. Transmission electron microscopy (TEM)

Standard Transmission Electron Microscopy (TEM) was employed to characterize the nanoparticles and their assemblies. Statistical distributions of particle sizes were obtained for each sample. Observations were performed at room temperature using a JEM2100F microscope (JEOL, Japan) equipped with a Schottky FEG gun, operating at 200 kV.

#### 4.1.3. Magnetometry

Magnetic properties of the samples were measured using a Magnetic Properties Measurement System (MPMS-XL). Hysteresis loops were recorded on frozen liquid samples over a field range from −2 T to 2 T, with a step size of 50 mT. The measurements were performed on a 20 μL drop of ferrofluid placed in an Eppendorf tube at 100 K. The diamagnetism contribution was removed by fitting the first 5 points of the magnetisation curve with a linear function.

### 4.2. Numerical

Molecular dynamics is a deterministic computer simulation technique used to study the dynamics of classical many-body systems. The basic idea of MD is to numerically integrate time-discretised Newton's equations of motion, for each particle in the simulation. In this work, however, we performed MD simulations based on the Langevin equations of motion, in the NVT ensemble. With this approach, the fast degrees of freedom (solvent) can be represented implicitly, as random forces. The Langevin equations of motion are given by:4
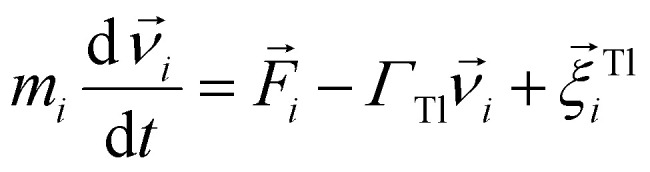
5
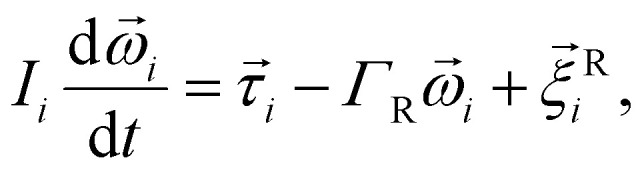
where for the *i*-th particle in eqn (4), *m*_*i*_ is the particle mass, 
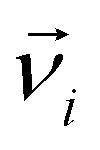
 denotes the translational velocity, 
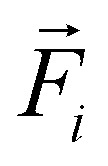
 is the force acting on it, *Γ*_Tl_ denotes the translational friction coefficient, 
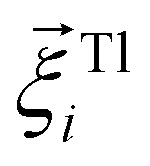
 is a stochastic force, modelling the random forces of the implicit solvent. In eqn (5), *I*_*i*_ denotes *i*-th particle moment of intertia, 
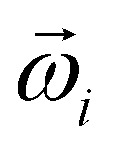
 is its rotational velocity, 
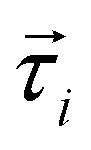
 is torque acting on it, *Γ*_R_ denotes the rotational friction coefficient, and the 
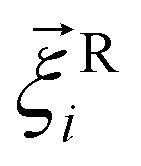
 is a stochastic torque serving the same purpose as 
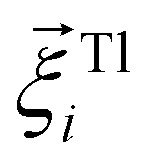
. The friction terms account for dissipation in a surrounding fluid, whereas the random force mimics collisions of the particles with solvent molecules at a fixed temperature. Both stochastic terms satisfy the following conditions on their time averages:^68^

6 〈*ξ*^Tl/R^_*l*_〉 = 0

7 〈*ξ*^Tl/R^_*l*_(*t*)*ξ*^Tl/R^_*k*_(*t*′)〉 = 2*Γ*_Tl/R_*k*_B_*Tδ*_*l*,*k*_*δ*(*t* − *t*′),

where *k*, *l* = *x*, *y*, *z*. Langevin equations of motion were integrated using the Velocity Verlet algorithm.^69^ We used periodic boundary conditions to simulate bulk sized systems. This means that along with the principal simulation box, where the particles are initially placed, an infinite number of identical replicas are created, so that when a particle leaves the principal box, its replica enters from a different side.^70^ All simulation work presented in this chapter was done using the ESPResSo simulation package.^71^

#### 4.2.1. Particles

The excluded volume of each spherical particle with diameter *σ* is achieved using the Weeks–Chandler–Andersen pair potential (WCA):^72^8
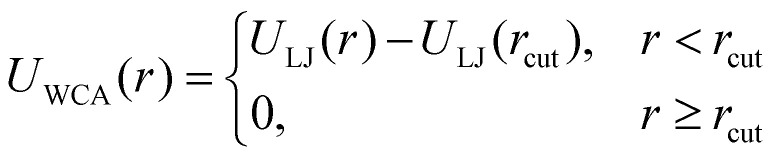
where *U*_LJ_(*r*) is the conventional Lennard-Jones potential:

9 *U*_LJ_(*r*) = 4*ε*{(*σ*/*r*)^12^ − (*σ*/*r*)^6^}

where *σ* is the characteristic diameter of the particle and the cutoff value is *r*_cut_ = 2^1/6^*σ*. The parameter *ε* defines the energy scale of the repulsion. Without any shift and a different cutoff (usually equal to several diameters *σ*), potential (9) can be used to describe central attraction in the Stockmayer approximation.

The dipole moments in MNPs were modelled as point dipoles, that interact *via* the dipole–dipole interaction potential:10
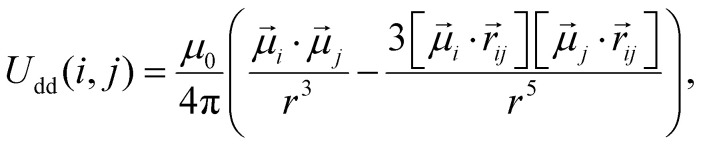
where 
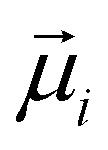
 and 
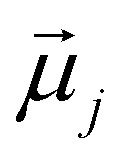
 are their respective dipole moments, 
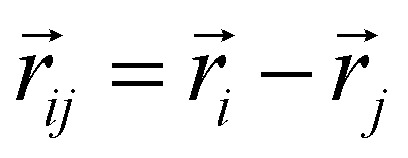
 is the displacement vector connecting their centres and 
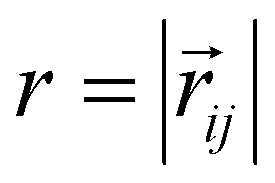
, *μ*_0_ is the vacuum permeability. The dipole–dipole interaction is a long-ranged interaction, and inducing a distance cutoff on it in simulations might lead to severe artifacts. To avoid this, magnetic interactions were calculated using the dipolar-P^3^M algorithm.^73^

In the presence of an external magnetic field 
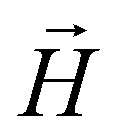
, each magnetic moment tends to coalign with its direction according to the Zeeman coupling potential:11
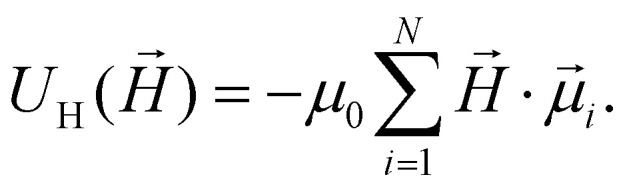


Magnetic degrees of freedom of MNPs were treated in different ways depending on their material type, as described below.

##### 4.2.1.1. Infinite anisotropy

In the simplest case, when the anisotropy of the MNP is very high, *κ* ≫ 1, one can ignore the internal degrees of freedom of the particle magnetisation and use the *fixed* point-dipole approximation. In this setup, the dipole moment rotates strictly with the physical rotation of the MNP body (Brownian relaxation).

##### 4.2.1.2. Zero anisotropy (ideal superparamagnet)

In case the magnetic anisotropy energy of the MNPs is vanishing, *κ* ≪ 1, we use the approach developed by Mostarac *et al.*^21^ The first step is to calculate the total field 
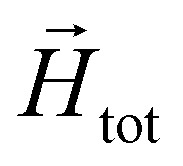
 in each point of the system. The total magnetic field is the sum of 
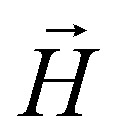
 and the dipole field 
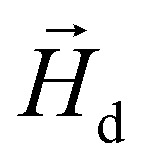
. The latter field, created by the magnetic particle *j*, at position 
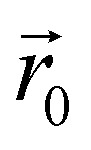
 is given by:12
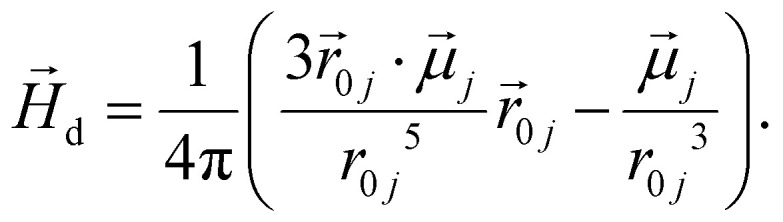


The study of the response of a filament to fields of arbitrary strength requires one to define the dipole moment, 
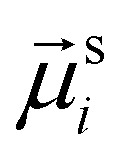
, of an *i*-th super-paramagnetic particle at a given temperature *T*, as:13
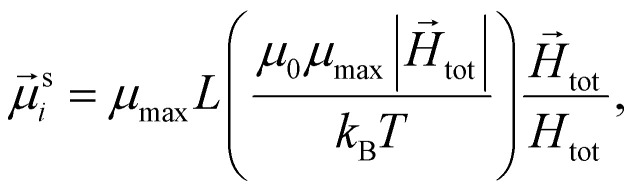
where 
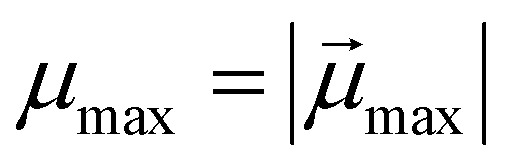
 denotes the modulus of the maximal magnetic moment of the particle, 
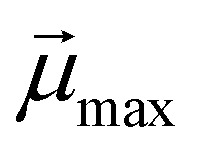
.

Here, *k*_B_ is the Boltzmann constant and *L*(*α*) is the Langevin function:14
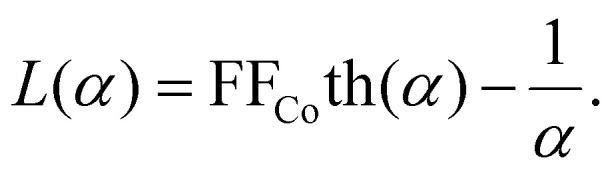


This method is inspired by the mean field theory,^74^ where the effective field acting on each magnetic dipole is estimated. Here, instead of estimation, we perform a direct calculation, taking into account non-linear effects. This approach is also verified by the analytical calculations for super-paramagnetic particles frozen in the matrix.^75^

##### 4.2.1.3. Finite anisotropy

For MNPs with *κ* ∼ 1, one must consider and simulate coupled rotational dynamics of particles and their magnetic moments. For this purpose, we used the so-called “egg model”. It was first introduced by Shliomis and Stepanov to describe the non-equilibrium magnetic response of dilute ferrofluids.^64^ In recent years, it has begun to gain increasing popularity as a robust tool to model superparamagnetic dynamics.^63,76–78^

In addition to eqn (4) and (5) describing the particle mechanical degrees of freedom, the egg model introduces one more equation for the dynamics of its magnetic moment. This equation is an overdamped Landau–Lifshitz–Gilbert equation written in a body-fixed frame of the particle (“overdamped” here means that the Larmor precession is neglected). In a laboratory reference frame, the equation then takes form15
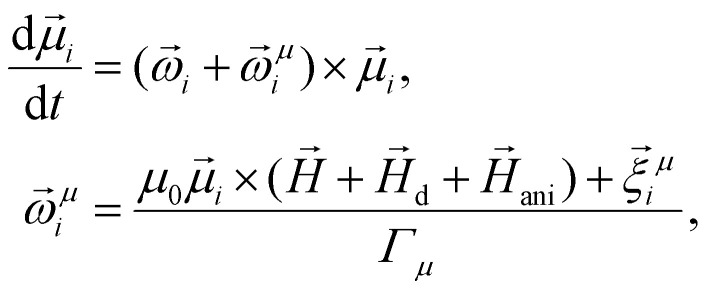
where *Γ*_*μ*_ and 
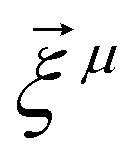
 are the “magnetic friction” coefficient and “magnetic noise”, respectively,

16 〈*ξ*^*μ*^_*l*_〉 = 0,

17 〈ξ^*μ*^_*l*_(*t*)ξ^*μ*^_*k*_(*t*′)〉 = 2*Γ*_*μ*_*k*_B_*Tδ*_*l*,*k*_*δ*(*t* − *t*′),

*H*_ani_ is the so-called anisotropy field. For a uniaxial anisotropy, considered in this work, it can be written as18
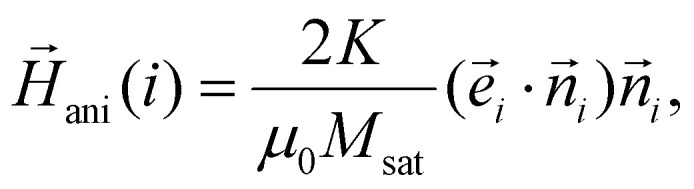
where *K* is the particle anisotropy constant introduced above, *M*_sat_ is its saturation magnetisation, 
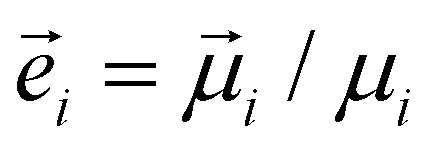
 is the unit vector along the particle magnetic moment, 
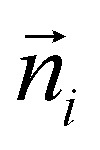
 is the unit vector along the particle anisotropy axis. The latter is rigidly connected to the particle body and rotates along with it.

Our ESPResSo implementation of the model neglects inertial terms on the left-hand sides of eqn (4) and (5) and uses the Euler–Maruyama algorithm instead of velocity Verlet. MNPs whose dipole moment was propagated using the egg model are referred to throughout the manuscript as egg model particles.

#### 4.2.2. Simulation protocol and parameters

In order to fix a characteristic length-scale in simulations, we fix the diameter of CoFe_2_O_4_ to *σ*_h_ = 1, following the experimental size ratio, we set the diameter of MnFe_2_O_4_ to *σ*_s_ = 1.3. Fixing both *ε* in eqn (9) and thermal energy *k*_B_*T* to unity, we rescale magnetic moments from experiments to obtain 
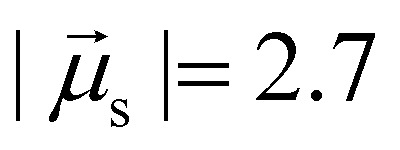
 and 
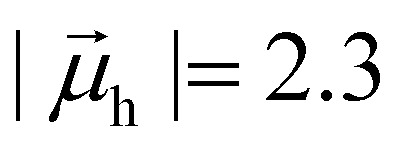
 for magnetically soft and hard particles correspondingly. Depending on the total volume fraction of magnetic material, *ϕ*, the simulation box length is fixed either to 17*σ*_h_ (*ϕ* = 0.1), or 80*σ*_h_ (*ϕ* = 0.001). These sizes are chosen for the number of particles in the less represented fraction to not be smaller than 100, while the total number of particles does not exceed 1000. Each simulation is initialised by specifying the number of CoFe_2_O_4_ and MnFe_2_O_4_ particles according to a selected value of *ϕ*_s_ and *ϕ*. The values of *κ* are respectively set to 18 and 3 at room temperature.

##### 4.2.2.1. Room temperature simulations in an implicit liquid carrier

CoFe_2_O_4_ particles are initialised with randomly oriented permanent magnetic dipole moments of fixed magnitude. Note that it can be done, as the anisotropy constant of those particles is so high that at room temperature in a liquid carrier the relaxation follows the Brownian mechanism, no matter if an external magnetic field is applied or not. MnFe_2_O_4_ particles are set as either egg model particles, or ideally superparamagnetic.

The binary mixture is first initialised by minimizing overlaps using a steepest descent algorithm. Thermal fluctuations are then introduced through a Langevin thermostat with fixed thermal energy and species-specific friction coefficients in eqn (4) and (5) for respectively (h)ard and (s)oft particles: *Γ*^h^_R_ = 35, *Γ*^h^_Tl_ = 105, *Γ*^s^_R_ = 77.5, *Γ*^s^_Tl_ = 137. If the egg model is employed, in eqn (15), *Γ*_m_ = 2. Following initial equilibration, dipolar interactions are enabled and the system is evolved using Brownian dynamics.

The main production run consists of 10^6^ integration steps with a time step *τ* = 0.0075, during which data is collected. This includes particle positions, dipole orientations, and the total system magnetisation. The choice of the time step allows resolving both internal dynamics and microscopic rotational dynamics of magnetically soft particles. An applied magnetic field, *H*, was chosen so that *μ*_0_*μ*_s_*H*/*k*_B_*T* ∼ 135; it corresponds to the saturation of the equilibrium magnetisation curve for any composition and *ϕ*.

As an outcome, for each *ϕ*–*ϕ*_s_ combination, six different simulations were performed: hard-egg (PDE) in zero applied field, hard-ideally superparamagnetic mixture (PDS) in zero applied field, PDE in an applied saturating field, PDS in an applied saturating field, PDE without dipole–dipole interactions in zero applied field, and noninteracting PDE under the influence of an applied saturation field.

Cluster analysis with energy-distance criteria^61^ is performed on 150 statistically independent snapshots for each binary mixture listed above. According to this criterion, two particles are bonded (clustered) if the distance between their centres does not exceed the distance at close contact by more than 30% and the magnetic interaction energy between particle dipoles is negative.

##### 4.2.2.2. Hysteresis loops of frozen samples

For creating frozen samples, 50 random statistically independent equilibrium configurations were selected and fixed in space. Both Brownian rotation and translation of particles were blocked, the value of thermal energy was reduced by a factor of three, as in the experiment, *k*_B_*T* = 0.3. It is worth saying that otherwise, the freezing protocol is not identical in simulations and experiments. In simulations, we perform instantaneous freezing like in ref. 79 featuring room-temperature aggregation and its influence on the magnetic response. It was done on purpose in order to understand the impact of structural transformations occurring during slow vitrification in the experiment. ZFC samples are obtained by freezing equilibrium configurations obtained in the absence of an applied magnetic field, while FC snapshots were taken from room temperature simulations with an applied saturating magnetic field.

CoFe_2_O_4_ particles are assigned finite anisotropy that is 6 times higher than for MnFe_2_O_4_. The maximum value of the field is chosen to be the same as at room temperature. Several field-steps and field changing frequencies were sampled, and the least computationally expensive parameters to assure equilibrium magnetisation process in every sample were chosen. As a result, on each quarter of the hysteresis loop, 50 field values were chosen. For each of these 200 field values, a 10^3^ equilibration steps and 4 × 10^3^ measurements were performed. The latter were averaged to obtain a point on a loop. For each frozen configuration, four magnetisation loops were performed, and the resulting 50 × 4 loops were finally averaged to obtain the resulting plots.

### 4.3. Theoretical

#### 4.3.1. Binary Stoner–Wohlfarth model

Hysteresis loop area eqn (2) in case of the mixture of magnetically soft (“s”) and hard (“h”) particles can be rewritten as19
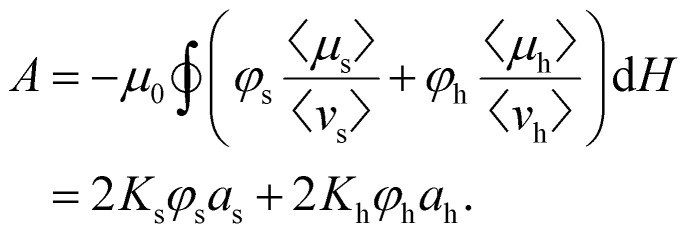


In this section, we will use index β to denote the particle type (*i.e.*, β = s or β = h). Thus, *μ*_β_ is the component of the particle magnetic moment along the field, *v*_β_ is the particle volume, 〈…〉 denotes averaging over the particle ensemble, *K*_β_ is particle anisotropy constant, and *a*_β_ is the dimensionless loop area of an individual mixture component,20
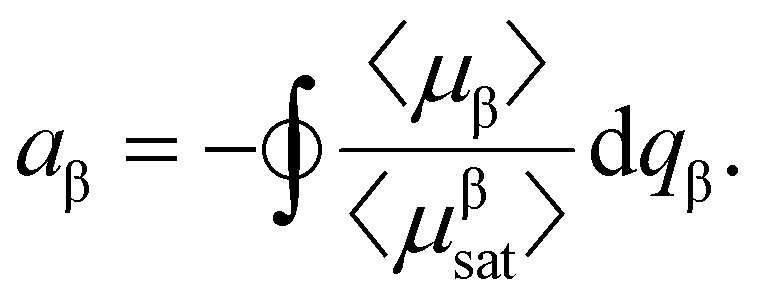


Here, *q*_β_ = *H*/*H*^β^_ani_ is the dimensionless magnetic field, *H*^β^_ani_ = 2*K*_β_/*μ*_0_*M*^β^_sat_ is the anisotropy field, *M*^β^_sat_ is the saturation magnetisation of the particle material, 〈*μ*^β^_sat_〉 = *M*^β^_sat_〈*ν*_β_〉. If one neglects interparticle interactions, integrals *a*_β_ will be defined by the magnetodynamics of individual particles and will not depend on the particle volume fractions. Then for a fixed overall particle content (*i.e.*, for *ϕ* = *ϕ*_s_ + *ϕ*_h_ = const), the dependency of *A* on *ϕ*_s_ must be *linear*:21
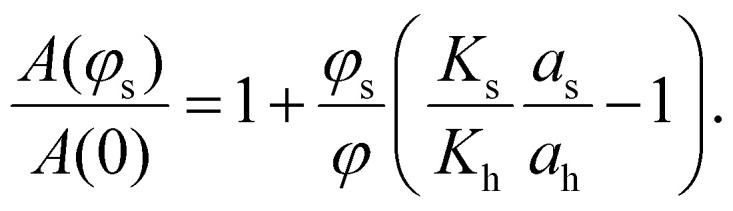


Upon additionally neglecting thermal fluctuations, particle polydispersity and assuming uniaxial magnetic anisotropy, one arrives at the classical Stoner–Wohlfarth model.^80^ Within its framework, *a*_β_ is solely defined by the orientational distribution of particles’ easy axes. Specifically, 0 ≤ *a*_β_ ≤ 4 – zero value corresponds to the orthogonal orientation of axes and the field (no hysteresis) while *a*_β_ = 4 corresponds to the perfect alignment of two (square-shaped hysteresis loop). To obtain eqn (3), one needs to further assume that *a*_s_ = *a*_h_, meaning that magnetically soft and hard particles have the same orientational distribution of easy axes (say, a random uniform distribution for ZFC scenario and a parallel texturing for FC one).

## Conflicts of interest

The authors declare no conflict of interest.

## Supplementary Material

NR-018-D5NR05218A-s001

## Data Availability

The data that support the findings of this study are available from the corresponding authors upon reasonable request. Supplementary information (SI): Fig. S1: Element-selective map of a chain formed by CoFe_2_O_4_ and MnFe_2_O_4_ mixed in a 1 : 1 ratio. (PDF). Fig. S2: Element-selective magnetisation curves measured by RIXS-MCD spectroscopy at Co and Mn edge in the single-phase and in the binary ferrofluid. (PDF). Fig. S3: Diameter distribution histogram of samples FF_Co_ and FF_Mn_. (PDF). Fig. S4: Representatives cryo-TEM images of pure FF_Co_, pure FF_Mn_, and 1 : 1 binary samples. Composite element-selective X-ray microscopy images. (PDF). Fig. S5: Probability heat maps for cluster sizes. (PDF). Fig. S6: Probability heat maps for number of neighbours. (PDF). Fig. S7: Magnetisation curves of binary ferrofluids at *φ* = 0.1 under ZFC conditions. (PDF). Fig. S8: Magnetisation curves of binary ferrofluids at *φ* = 0.1 under ZFC conditions without interactions. (PDF). See DOI: https://doi.org/10.1039/d5nr05218a.
