# Supplementary material for: Hard meets soft: tuning binary ferrofluids
Source: Nanoscale. 2026 Mar 6;18(22):11724–38. doi: 10.1039/d5nr05218a (PMC12998614; doi:10.1039/d5nr05218a)
Supplement: NR-018-D5NR05218A-s001 [file NR-018-D5NR05218A-s001.pdf]

Supplementary Information (SI) for Nanoscale.
This journal is © The Royal Society of Chemistry 2026

Supporting Information:

Hard Meets Soft: Tuning Binary Ferrofluids

Malika Khelfallah,[*,†] Ekaterina V. Novak,[‡] Andrey A. Kuznetsov,[¶] Deniz Mostarac,[¶] Niéli Daffé,[†,§,‖] Marcin Sikora,[⊥] Sophie Neveu,[§] Jovana Zečević,[#] Johannes D. Meeldijk,[#] Dario Taverna,[†] Philippe Sainctavit,[†] Mauro Rovezzi,[@,△] Hebatalla Elnaggar,[†] Enzo Bertuit,[†,§] Nicolas Mille,[‖] Rachid Belkhou,[‖] Vincent Dupuis,[§] Claire Carvallo,[†] Amélie Juhin,[*,†] and Sofia S. Kantorovich[*,¶]

†*IMPMC, CNRS UMR7590, Sorbonne Université, MNHN, 4 Place Jussieu, Paris, France*

‡*Ural Federal University, Lenin Av. 51, Ekaterinburg 620000, Russian Federation*

¶*Faculty of Physics, University of Vienna, Boltzmanngasse 5, 1090 Vienna, Austria*

§*Sorbonne Université, CNRS, PHysicochimie des Électrolytes et Nanosystèmes InterfaciauX, PHENIX, F-75005 Paris, France*

‖*Synchrotron SOLEIL, L'Orme des Merisiers, Saint-Aubin – BP48, 91192 Gif-sur-Yvette, France*

⊥*AGH University of Science and Technology, Academic Centre for Materials and Nanotechnology, Al. Mickiewicza 30, 30-059 Krakow, Poland*

#*Inorganic Chemistry and Catalysis, Debye Institute for Nanomaterials Science, Utrecht University, Universiteitsweg 99, 3584 CG Utrecht, The Netherlands*

@*European Synchrotron Radiation Facility (ESRF), 6 Rue Jules Horowitz, BP220, 38043 Grenoble Cedex 9, France*

△*Univ. Grenoble Alpes, CNRS, IRD, Irstea, Météo France, OSUG, FAME, 38000 Grenoble, France*

E-mail: malika.khelfallah@gmail.com; amelie.juhin@sorbonne-universite.fr; sofia.kantorovich@univie.ac.at

Preliminary experimental results on different $CoFe_2O_4$ - $MnFe_2O_4$ systems

In this section we present experimental results obtained on $CoFe_2O_4$ - $MnFe_2O_4$ binary ferrofluids that are slightly different from the system investigated in the paper.

Element-selective microscopy (cryo-TEM EDX) on a 1:1 binary ferrofluid of $CoFe_2O_4$ (25 nm) - $MnFe_2O_4$ (15 nm) nanoflowers

Experimental details

Cryogenic Transmission Electron Microscopy (cryo-TEM) experiments coupled to Energy Dispersive X-ray (EDX) analysis were conducted at the Debye Institute for Nanomaterials Science in Utrecht (Netherlands). The specimen was prepared by vitrification, i.e. a drop of ferrofluid was rapidly frozen in liquid ethane to prevent aggregation or spatial reconfiguration of the positions of the nanoparticles inside the fluid. More precisely, 3 μL of binary ferrofluid sample were drop-casted on a holey carbon TEM grid and plunge-frozen in liquid ethane using a Vitrobot (ThermoFischer). The grid was transferred under liquid nitrogen to cryo-TEM holder (Gatan 626 side entry cryo-holder), and 2k $\times$ 2k px STEM images were collected using a Talos™ F200X (ThermoFischer) microscope operating at 200 kV. Elemental EDX maps were acquired using the same microscope equipped with Super-X G2 EDX detector and Velox™ analytical and imaging software, at 200 kV. About 300 frames of 512 $\times$ 512 px with dwell time of 5 μs were recorded per EDX map, amounting to 6–7 min of acquisition time per map.

Cryo-TEM EDX results

Figure S1(a) shows a STEM image of a vitrified binary ferrofluid composed of $CoFe_2O_4$ nanoflowers (25 nm in diameter) and $MnFe_2O_4$ nanoflowers (15 nm in diameter) mixed in a

1:1 ratio. In Figure S1(b) the chemical composition of nanoparticle-flowers, as measured by EDX, is shown. The larger nanoparticle-flowers forming a chain are $CoFe_2O_4$ nanoflowers, while the $MnFe_2O_4$ nanoflowers are located on the sides of the chains.

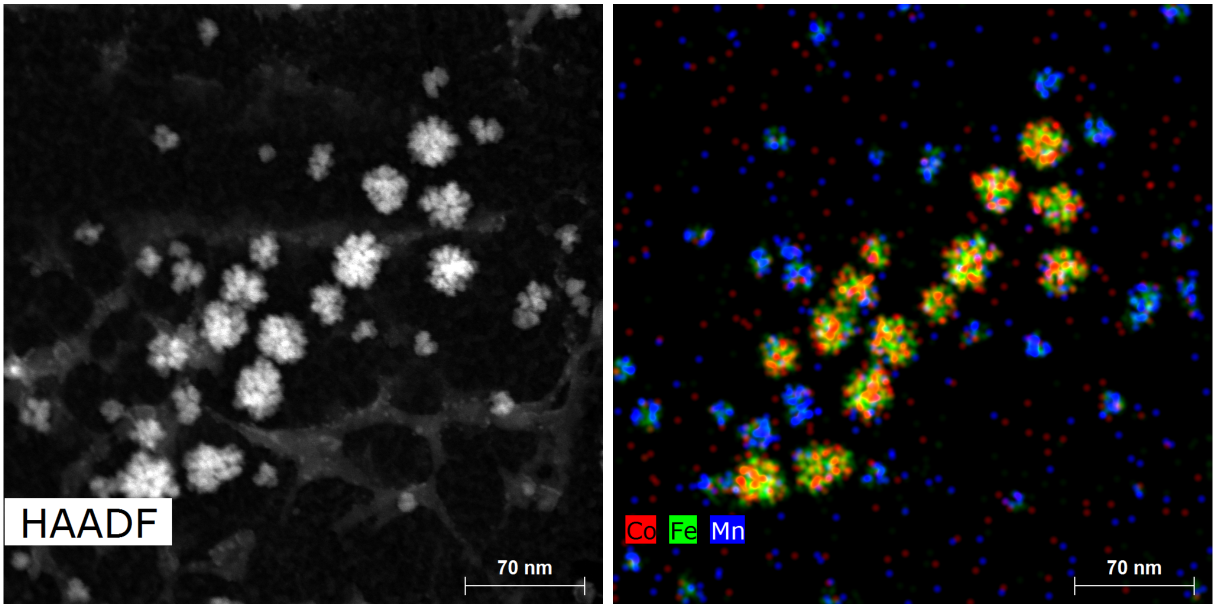

(a) (b)

Figure S1: Element-selective map of a chain formed by $CoFe_2O_4$ and $MnFe_2O_4$ mixed in a 1:1 ratio. (a) HAADF image of a typical region of the sample. (b) Corresponding chemical map showing the elemental distribution of Co (red), Fe (green) and Mn (blue).

Element-selective magnetisation curves in a binary ferrofluid of $CoFe_2O_4$ (25 nm) - $MnFe_2O_4$ (20 nm) nanoflowers

Experimental details

Element-selective magnetisation curves were measured using RIXS-MCD spectroscopy, which couples Resonant Inelastic X-ray Scattering and Magnetic Circular Dichroism.[S1]

RIXS-MCD experiments were carried out at ID26 beamline of the European Synchrotron Radiation Facility (Grenoble, France). Measurements were performed at the Co and Mn K-edges at low temperature (30 K) on the frozen phase of ferrofluids using a dedicated liquid cell, in the same experimental conditions as in Ref. S1. The uncertainty on temperature was

estimated to be ±5 K. Samples were frozen either without an external magnetic field (ZFC) or with a fixed external magnetic field of 1.5 T (FC) delivered by an electromagnet.

1s2p RIXS and RIXS-MCD planes were first measured across the K pre-edge region (first Co, then Mn) in order to determine the values of incident and emission energies that maximize the magnetic contrast. Element selective magnetisation curves were then measured by fixing energies at these optimized values, and by sweeping the external magnetic field between 1.5 T and -1.5 T and back to 1.5 T. More details can be found in Ref. S1.

RIXS-MCD results

Element-selective magnetisation curves measured at the Co and Mn edges were measured on both monocomponent ferrofluids ($CoFe_2O_4$ (25 nm) nanoflowers and $MnFe_2O_4$ (20 nm) nanoflowers) and a $CoFe_2O_4$-$MnFe_2O_4$binary ferrofluid (1:1 ratio, $\varphi = 0.003$). At the Co edge, Fig. S2(a) shows hardly no difference between the magnetisation curve of the $CoFe_2O_4$ reference and that of the $CoFe_2O_4$ component in the binary ferrofluid. In Fig. S2(b), the M(H) curve is measured in the binary ferrofluid under zero field cooled and field cooled conditions. The magnetic properties are modified and we observe an increase of coercivity for the $CoFe_2O_4$ component is observed in the FC conditions. Finally, the magnetisation curve measured at the Mn edge is presented in Fig. S2(c). The slope and remanence of the hysteresis are decreased for the $MnFe_2O_4$ component in the binary ferrofluid with respect to the pure phase, while the coercivity is not modified.

Structural properties of the 1:1 binary ferrofluid of $CoFe_2O_4$ (15nm) - $MnFe_2O_4$ (20nm) nanoflowers

Here are presented additional microscopy results measured on the 1:1 binary ferrofluid of the paper.

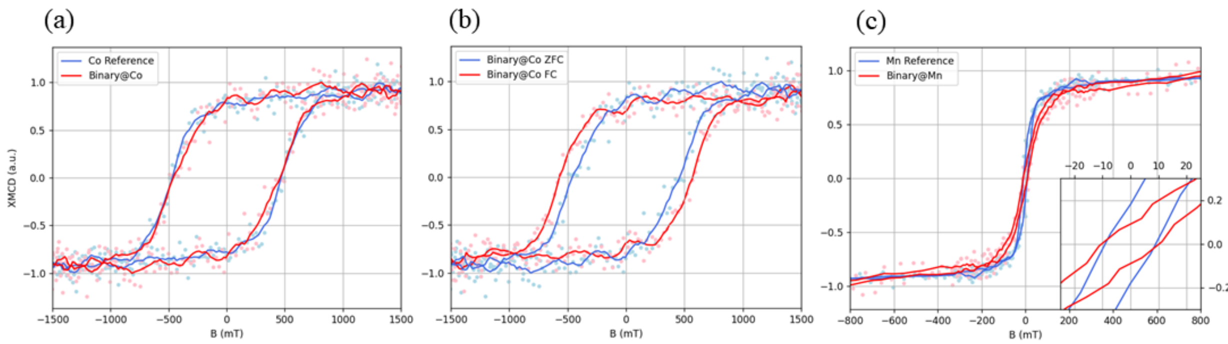

Figure S2: Element-selective magnetisation curves measured by RIXS-MCD spectroscopy at (a) Co edge in the single-phase ($CoFe_2O_4$ reference) ferrofluid and in the binary ferrofluid, (b) Co edge in the binary ferrofluid under field cooled (FC) and zero field cooled (ZFC) conditions, and (c) Mn edge in the single-phase ($MnFe_2O_4$ reference) ferrofluid and in the binary ferrofluid.

Experimental details

Cryo-TEM

Cryo-TEM experiments were conducted at IMPMC in Paris (France). Cryo-TEM sample grids were prepared using an automatic cryo-plunger (LEICA, Germany). A 5 μL drop of the ferrofluid was deposited on "Quantifoil"® carbon membrane grids (Quantifoil Micro Tools GmbH, Germany), which were plasma-cleaned for 30 seconds. Excess liquid was removed by blotting the grid with filter paper for three seconds, and the grid was rapidly frozen in liquid ethane, forming a thin vitreous ice film. The cryo-holder (GATAN, USA) maintained the samples at low temperatures (−180 °C) for observation with a JEM2100 microscope (JEOL, Japan) equipped with a LaB6 electron gun operating at 200 kV. Cryo-TEM images were recorded using an Ultrascan 1000, $2k \times 2k$ pixels CCD camera (Gatan, USA).

STXM

Scanning Transmission X-ray Microscopy (STXM) experiments were conducted at the HERMES beamline of the SOLEIL synchrotron. Chemical mapping of the assemblies was achieved by illuminating the samples at specific energies corresponding to the Co, Mn, and Fe absorption edges at room temperature. For STXM sample preparation, TEM grids were plasma-

cleaned under an argon atmosphere for 30 seconds, transforming the carbon membrane into a hydrophilic surface through the introduction of hydroxyl (-OH), carboxyl (-COOH), and carbonyl (-CO) groups, while removing contaminants. A 5 μL drop of diluted ferrofluid $\varphi \sim 0.001$ was deposited on the prepared grid held with reverse tweezers. The grid was then left to dry in air for 12 hours. To achieve a field-cooled (FC) configuration, the grid was placed between two permanent magnets during the drying phase. The magnetic field lines were aligned parallel to the grid plane, and the field strength was calibrated using a Hall probe. Adjustments to the magnetic field strength were made by varying the distance between the magnets.

Microscopy results

Single-phase ferrofluids as well as binary mixtures with 1:1, 1:2 and 3:1 ratios were analysed to investigate the chemical and spatial distribution of the nanoparticles within the assemblies. In Fig. S3,

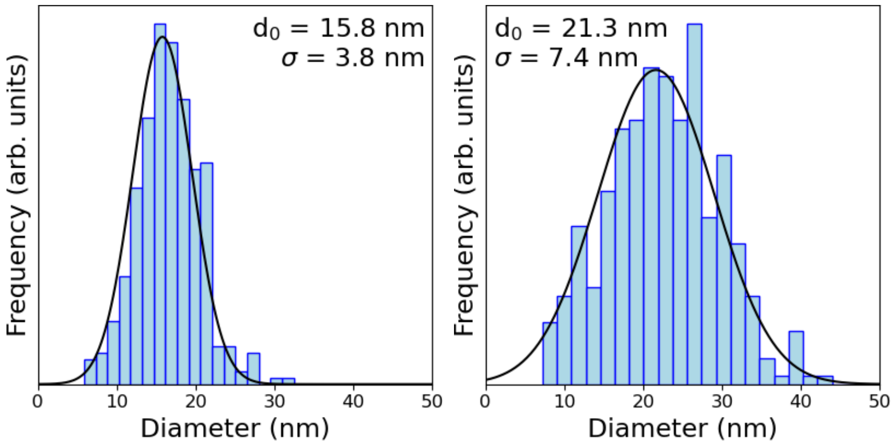

(a) (b)

Figure S3: Diameter distribution histogram of samples (a) FF_{Co} and (b) FF_{Mn} fitted with a gaussian function.

In Fig. S4 one can get a sense for the structural organisation of nanoparticles in the absence of an external magnetic field from representative cryo-TEM images of monocomponent and binary ferrofluid samples.

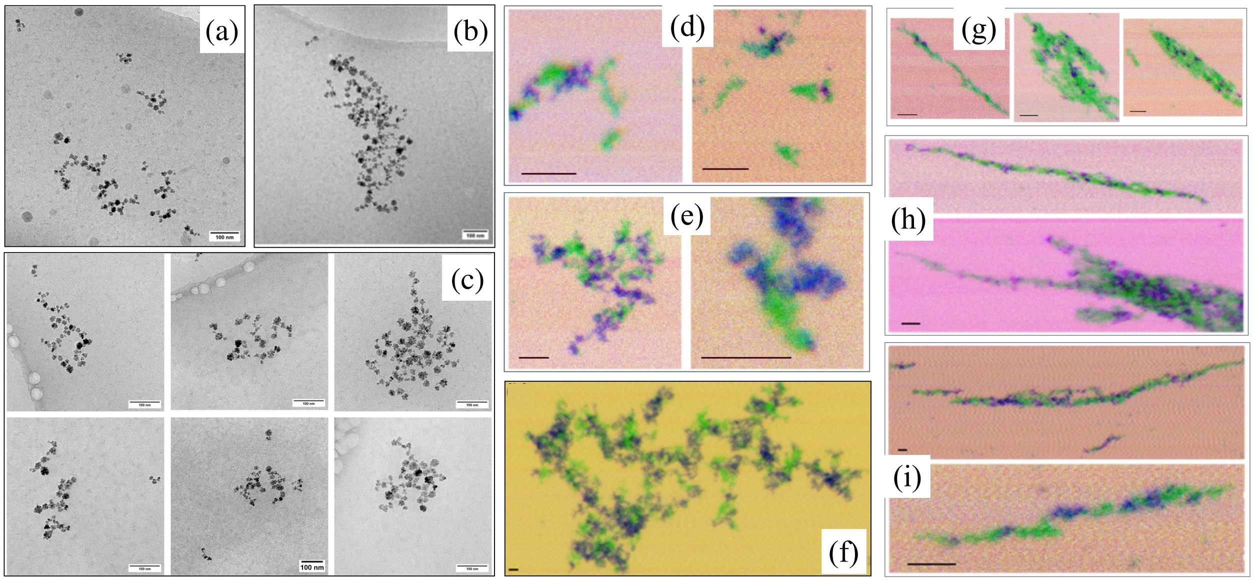

Figure S4: Representatives cryo-TEM images of FC (a) pure FF_{Co}, (b) pure FF_{Mn}, and (c) 1:1 binary samples. Subfigure (c) contains 6 images. Total volume fraction of nanoparticles is the same across the samples, $\varphi = 0.001$. The scale-bar is 100 nm. Composite element-selective X-ray microscopy images. Green: $CoFe_2O_4$; blue/purple: $MnFe_2O_4$. (d)–(f) ZFC samples; (g)–(i) FC samples. Volume ratios of $CoFe_2O_4$:$MnFe_2O_4$ are (d), (g) 3:1, (e), (h) 1:1, and (f), (i) 1:2. Scale bar: 200 nm.

In the case of the pure cobalt ferrite sample (FF_{Co}, Fig. S4(a)), the nanoparticles form small, compact clusters, and short chain-like structures. Moreover, the observed chaining behavior suggests that, on cooling, the magnetic energy is sufficient to overcome thermal agitation and induce directional self-assembly during vitrification, even in dilute suspensions (volume fraction $\varphi = 0.001$). The cryo-TEM specimen preparation can locally alter the structure due to the confinement of the fluid in a 2D layer on the TEM grid; thus, the local volume fraction of vitrified samples is higher than that of bulk liquid samples.

By contrast, the pure manganese ferrite ferrofluid (FF_{Mn}, Fig. S4(b)) displays a very different morphology. The soft magnetic $MnFe_2O_4$ nanoparticles tend to form large, irregularly shaped, branched, sparse aggregates comprising hundreds of particles.

Finally, in Fig. S4(c), for the binary ferrofluid, the structure is a combination of the two previous observations, incorporating both chains and clusters. However, chemical identification is not possible, and the composition of the clusters/chains cannot be determined.

To resolve the individual contributions of $CoFe_2O_4$ and $MnFe_2O_4$ nanoflowers within the binary assemblies, STXM, which is an element-selective spectromicroscopy, was employed.

Binary ferrofluid samples with FF_{Co}:FF_{Mn} ratios of 3:1, 1:1, and 1:2 were prepared, with and without the application of an external magnetic field. For each composition, 6–7 representative zones were imaged. Fig. S4 (d)–(i) shows selected images for both the ZFC and FC configurations. In the ZFC state shown in Figs. S4(d)–(f), nanoparticles form clusters comprising several hundred particles. These clusters contain both FF_{Co} and FF_{Mn} nanoflowers, although their spatial distribution is inhomogeneous. At a 3:1 ratio in Fig. S4(d), clusters predominantly consist of FF_{Co} nanoparticles with minor FF_{Mn} inclusions. In the 1:1 case (Fig. S4(e)), 100–200 nm clusters of like composition coalesce into micron-sized aggregates. For the 1:2 ratio presented in Fig. S4(f), elongated aggregates up to 8 µm are composed of distinct FF_{Co} or FF_{Mn} sub-clusters.

Upon field cooling, nanoparticles align into chains as evidenced by Figs. S4(g)–(i). At 3:1 shown in Fig. S4(g), these chains are primarily composed of FF_{Co} nanoparticles, with sparse FF_{Mn} inclusions acting as local defects. In the 1:1 system (see, Fig. S4(h)), chains extend up to 8 µm, with FF_{Co} dominating due to their larger volume fraction. For the 1:2 volume fraction ratio presented in Fig. S4(i), FF_{Mn} forms more prominent segments within the chains. The applied magnetic field promotes anisotropic head-to-tail alignment along the chain axis, generating structured environments distinct from single-component ferrofluids. The magnetically hard FF_{Co} nanoparticles enforce stability, while the FF_{Mn} nanoparticles undergo reorientation under the local field. Consequently, FF_{Mn} becomes magnetically harder, exhibiting increased coercivity due to interaction with FF_{Co}, or conversely, FF_{Mn} inclusions soften the otherwise magnetically hard FF_{Co}.

While cryo-TEM and element-selective STXM in Figs. S4 (a)–(i) offer essential insights into the spatial organization and chemical identity of nanoparticle assemblies in binary ferrofluids, these techniques are experimentally demanding and inherently limited in statistical reach due to constraints on sample preparation, imaging time, and field of view. Moreover,

cryo-TEM is performed on vitrified samples, which may not fully represent the dynamic structures present in the bulk, where direct visualization is even more challenging or altogether inaccessible.

Simulations results

Figure S5 presents probability heat maps for cluster sizes as a function of the volume fraction of soft particles, under two interaction models: the egg model (panels (a)–(c)) and the superparamagnetic model (panels (d)–(f)), with the total nanoparticle volume fraction fixed at $\varphi = 0.001$. In the egg model, mixtures of magnetically hard ($CoFe_2O_4$) and soft ($MnFe_2O_4$) particles (panel (a)) exhibit a decreasing probability of forming dimers as the volume fraction of soft particles increases. As panel (c) shows, soft particles remain non-aggregated. This suggests that $CoFe_2O_4$ particles play a dominant role in dipolar coupling and cluster formation. In contrast, the superparamagnetic model predicts negligible clustering across all compositions. Panels (d)–(f) show that, regardless of the soft particle concentration.

Figure S6 extends this analysis by examining the probability for a particle to have a certain amount of neighbours. In the egg model (panels (a)–(c)), hard particles are more likely to have one neighbour at low soft-particle concentrations, whereas soft particles remain mostly isolated (zero neighbours). As the soft fraction increases, connectivity decreases, consistent with reduced clustering ability. In the superparamagnetic model (panels (d)–(f)), vertex degrees remain low across all compositions, in line with the absence of significant clustering observed in Fig. S5.

Figure S7 presents the magnetisation curves (dashed lines) of binary ferrofluids at $\varphi = 0.1$ under ZFC conditions, where only the soft particles are allowed to remagnetise (the anisotropy of hard particles is set to infinity). Results are shown for $\varphi_s = 0.025$ (orange) and $\varphi_s = 0.067$ (violet). In this scenario, the magnetic response is entirely dominated by the soft component, while the hard particles remain magnetically frozen. The loops are shown

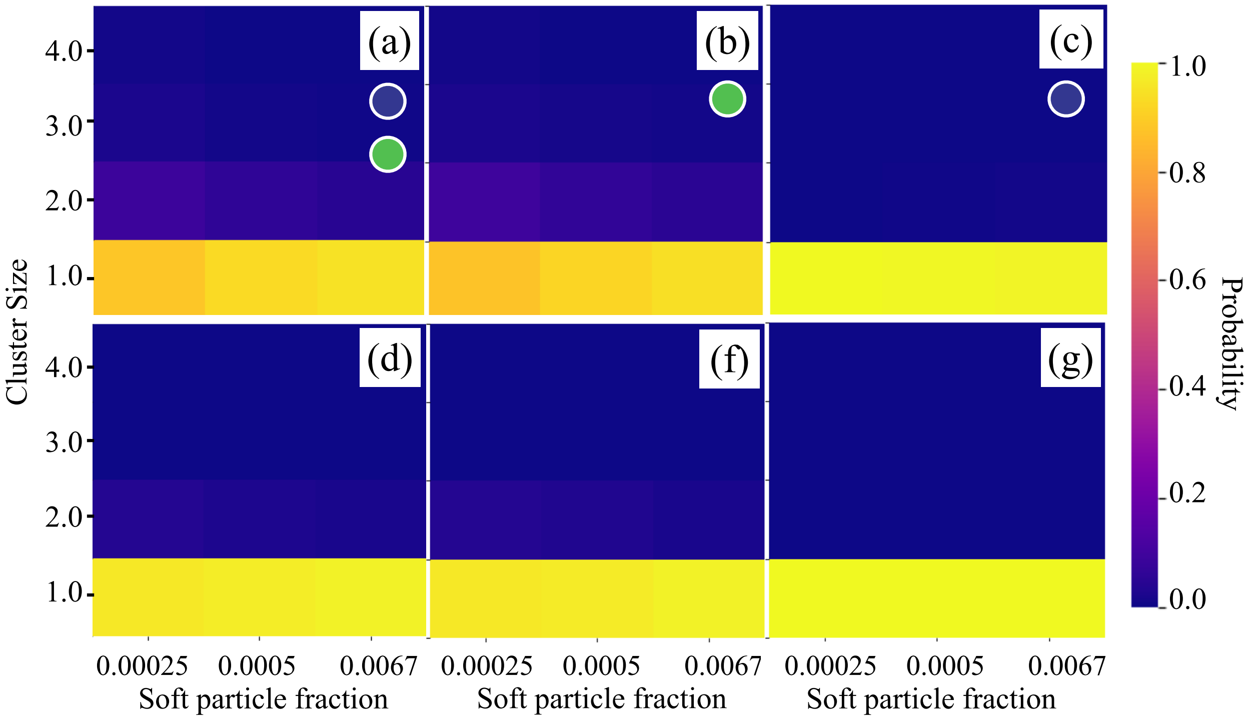

Figure S5: Probability heat maps for cluster sizes. (a)-(c) egg model: (a) Probability of finding clusters of specific sizes at different volume fractions for mixtures of soft and hard particles; (b) Probability of finding clusters of specific sizes at different volume fractions exclusively for hard particles; (c) Probability of finding clusters of specific sizes at different volume fractions exclusively for soft particles; (d)-(f) superparamagnetic model: (d) Probability of finding clusters of specific sizes at different volume fractions for mixtures of soft and hard particles; (e) Probability of finding clusters of specific sizes at different volume fractions exclusively for hard particles; (f) Probability of finding clusters of specific sizes at different volume fractions exclusively for soft particles. Total volume fraction is set at 0.001.

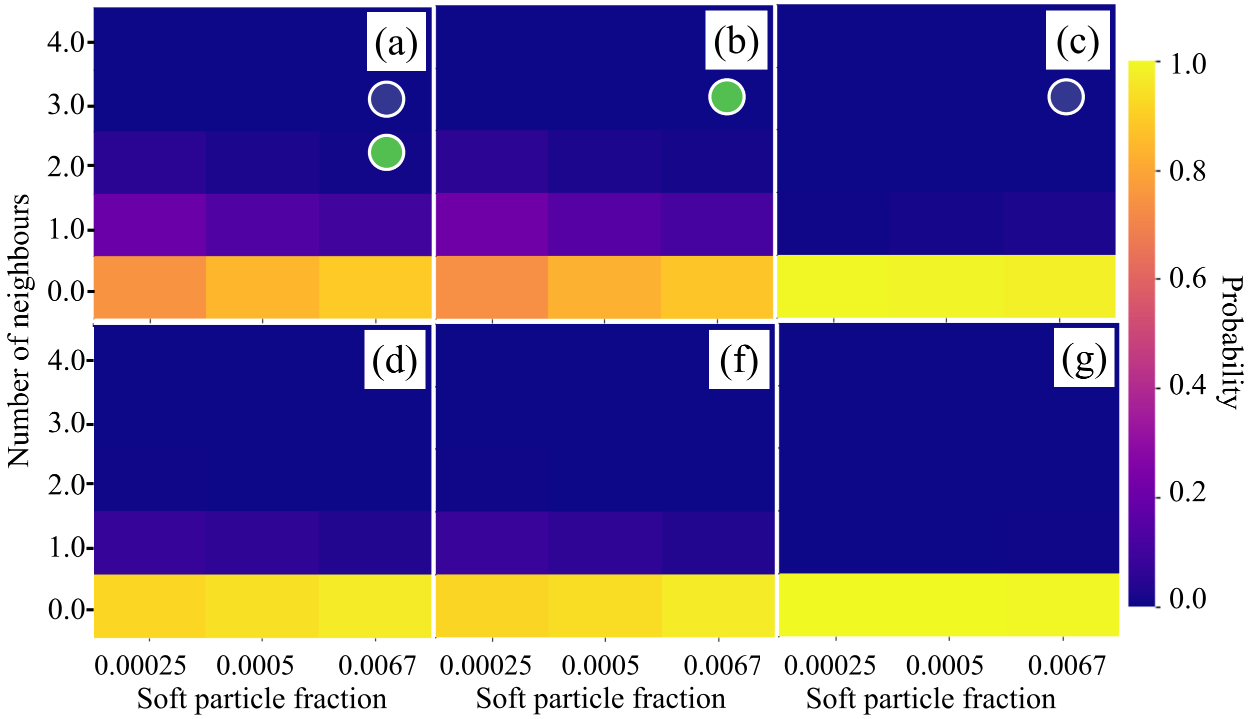

Figure S6: Probability heat maps for number of neighbours. (a)-(c) egg model: (a) Probability of detecting vertices with specific degrees in particle networks at different volume fractions for mixtures of soft and hard particles; (b) Probability of detecting vertices with specific degrees in particle networks at different volume fractions exclusively for hard particles; (c) Probability of detecting vertices with specific degrees in particle networks at different volume fractions exclusively for soft particles; (d)-(f) superparamagnetic model: (d) Probability of detecting vertices with specific degrees in particle networks at different volume fractions for mixtures of soft and hard particles; (e) Probability of detecting vertices with specific degrees in particle networks at different volume fractions exclusively for hard particles; (f) Probability of detecting vertices with specific degrees in particle networks at different volume fractions exclusively for soft particles; Total volume concentration is set at 0.001.

without normalisation. The solid lines correspond to the same systems, but with $CoFe_2O_4$ particles having finite magnetic anisotropy. It can be observed that, when the fraction of soft particles is small, the hysteresis is largely determined by the hard particles. In contrast, at higher soft-particle volume fractions, interactions between hard and soft particles, as well as between soft particles themselves, become significant, leading to a small hysteresis even for the soft particles, whose effective magnetic anisotropy is enhanced.

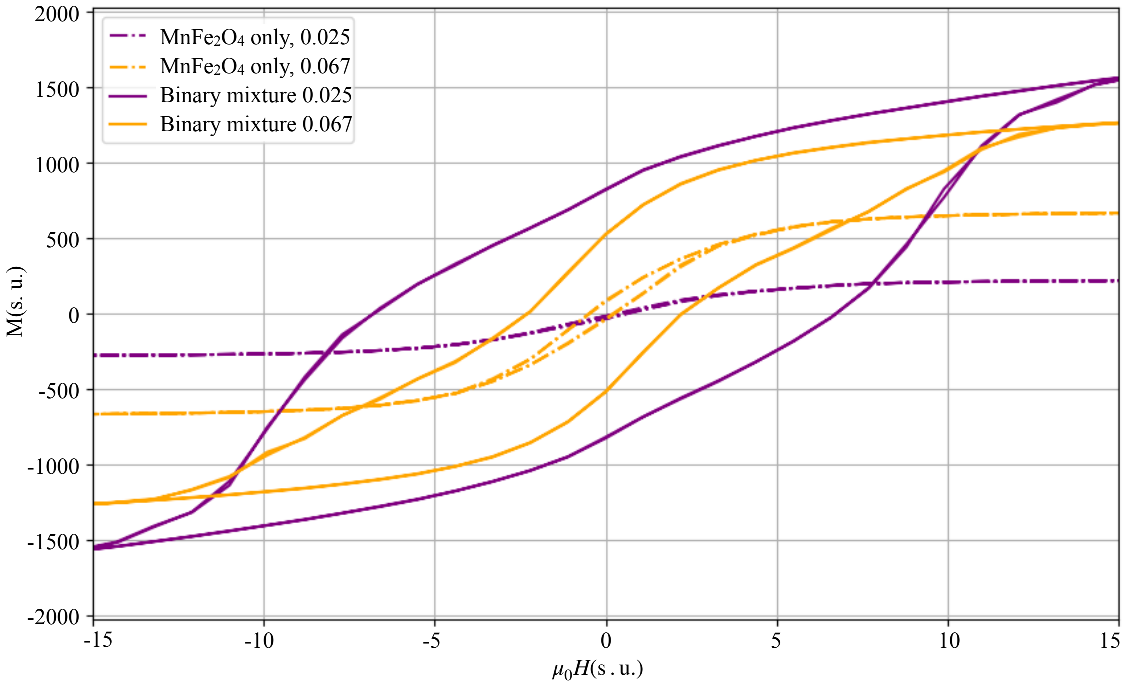

Figure S7: Magnetisation curves of binary ferrofluids at $\varphi = 0.1$ under ZFC conditions. Dashed lines: only soft particles remagnetise ($\varphi_s = 0.025$ orange, $\varphi_s = 0.067$ violet), hard particles frozen. Solid lines: same systems as in the main text loops for binary mixtures, where both hard and soft particles have finite magnetic anisotropy.

Finally, Figure S8 illustrates magnetisation curves for the same system at $\varphi = 0.1$ under ZFC conditions when all magnetic interactions are switched off (Stoner-Wohlfart model, dashed lines). Full simulation loops are shown with solid lines. Dipolar interactions clearly lead to the growth of the area of the hysteresis loops and of the coercive fields.

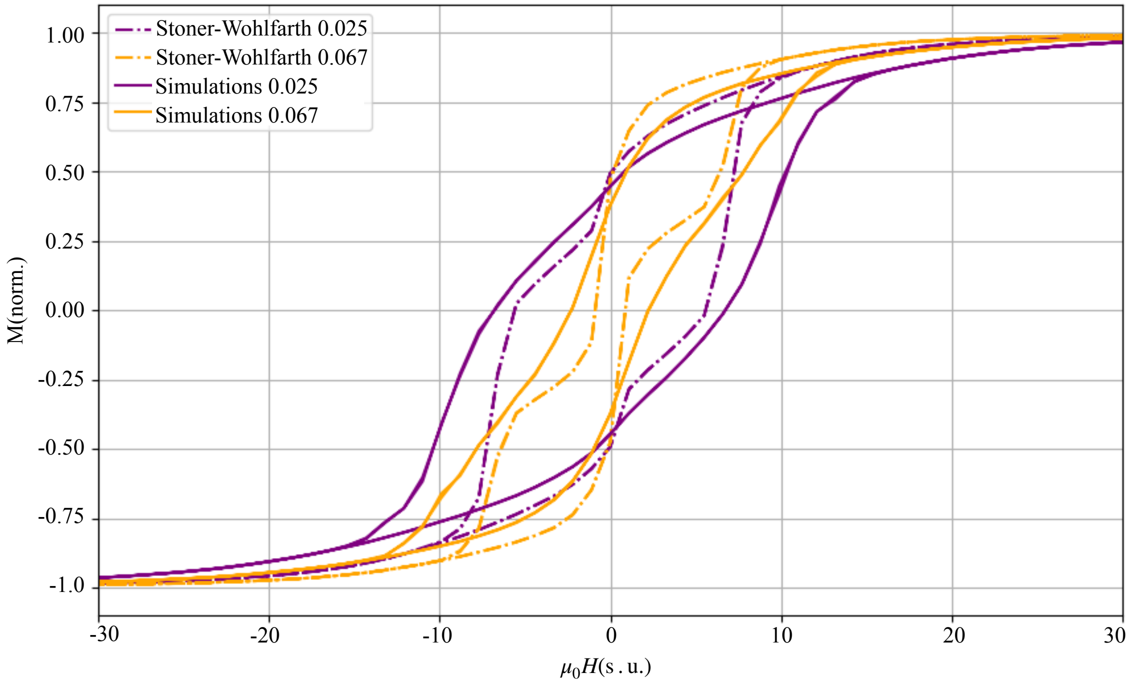

Figure S8: Magnetisation curves of binary ferrofluids at $\varphi = 0.1$ under ZFC conditions. Dashed lines: Stober-Wohlfart models, all magnetic interactions are switched off ($\varphi_s = 0.025$ orange, $\varphi_s = 0.067$ violet). Solid lines: same systems as in the main text loops for binary mixtures, where both hard and soft particles have finite magnetic anisotropy and interact.

References

(S1) Daffé, N.; Zečević, J.; Trohidou, K. N.; Sikora, M.; Rovezzi, M.; Carvallo, C.; Vasilakaki, M.; Neveu, S.; Meeldijk, J. D.; Bouldi, N.; Gavrilov, V.; Guyodo, Y.; Choueikani, F.; Dupuis, V.; Taverna, D.; Sainctavit, P.; Juhin, A. Bad Neighbour, Good Neighbour: How Magnetic Dipole Interactions between Soft and Hard Ferrimagnetic Nanoparticles Affect Macroscopic Magnetic Properties in Ferrofluids. *Nanoscale* **2020**, *12*, 11222–11231.
